# Encapsulating commercial accelerometers with epoxy and fluoroelastomer for harsh hydrocarbon fluid environment

**DOI:** 10.1038/s41598-023-46781-x

**Published:** 2023-11-13

**Authors:** Sahil P. Wankhede, Xian Du, Keith W. Brashler, Mohammad M. Ba’adani, Doru C. Turcan, Ali H. Shehri, Kamal Youcef-Toumi

**Affiliations:** 1 Department of Mechanical Engineering, University of Massachusetts Amherst, Amherst, MA 01003 USA; 2 Center for Personalized Health Monitoring, (CPHM), Institute for Applied Life Sciences (IALS), Amherst, MA 01003 USA; 3 Saudi Arabian Oil Company (Saudi Aramco), 31311 Dhahran, Saudi Arabia; 4 Massachusetts Institute of Technology, 77 Massachusetts Avenue, Cambridge, MA 02139 USA

**Keywords:** Chemistry, Polymer chemistry, Engineering, Mechanical engineering, Materials science, Materials for devices

## Abstract

Traditionally, in the oil and gas industry, accelerometers are mounted externally on motors for condition monitoring of vertically suspended, closed suction hydrocarbon pumps due to their inability to withstand harsh downhole environments, preventing the detection of impeller failures. This study addresses the need for encapsulation solutions for accelerometers submerged in hydrocarbon fluid environments. It evaluates the feasibility of epoxy and fluoroelastomer as encapsulation materials for long-term immersion in high-temperature hydrocarbon fluid and determines their impact on the accelerometer's performance. Extensive testing involved submersion in high-temperature hydrocarbon fluid at 150 °C for over 10,000 h and six months in brine. Material characterization, including mass variation, microscopic imaging, and FTIR spectroscopy, revealed negligible degradation. Encapsulated accelerometers effectively detected vibrations with an acceptable alteration in amplitude. In comparison with commercial alternatives, our encapsulation outperformed them. While oil traces became evident within just 24 h in the alternatives, our solution exhibited no signs of leakage. This research pioneers a novel packaging solution employing epoxy and fluoroelastomer for side-exit commercial sensors tailored for high-temperature hydrocarbon fluid applications, addressing a critical gap in the industry. Our work enhances reliability and safety for vertical oil pump condition monitoring in downhole applications, benefiting the oil and gas sector.

## Introduction

Vertically suspended, closed-suction hydrocarbon pumps in the oil and gas industry are vital components to support oil production. Effectively monitoring the condition of these pumps utilizing new technologies such as embedded sensors for IoT condition monitoring^1^ digital twin modeling^2,3^ and machine learning methods^4,5^ allows for the early detection of potential failure modes that occur at the working end or 1st stage impeller location. This proactive approach not only streamlines maintenance planning but also results in significant cost reductions and minimized instances of unplanned maintenance. It shifts the focus towards condition-based maintenance, as opposed to relying solely on fixed time intervals. Furthermore, it enhances operational safety and contributes to the extended lifespan of oil pumps, achieved through well-informed maintenance decisions based on real-time sensor data.

Traditionally vibration measurement for condition monitoring was conducted on the motor outside the oil well using accelerometers^6^. However, this method does not have the sensitivity for early detection of typical failures that occur underground at the 1st-stage impeller location in the Aramco oil fields, which cannot be easily accessible for daily or periodic maintenance schedules similar to the entire machinery/structure^7^. For instance, in the oil and gas industry oil pumps are submerged in the oil well and subjected to a maintenance period of at least 2 years^8^.

To address this limitation, an accelerometer can be mounted near the 1st-stage impeller to detect failure modes early. However, mounting an accelerometer at this location is difficult because of fluid compatibility, safety, reliability, and accessibility. Furthermore, they might be exposed to harsh environments like crude oil/hydrocarbons/petroleum, high temperature, humidity, salinity, severe radiation, or a mix of these conditions. Therefore, most of the time accelerometers are mounted on the pump discharge head or motor which makes it difficult to detect failure modes occurring at the wet-end of the pump for monitoring^6^.

**Figure 1:**
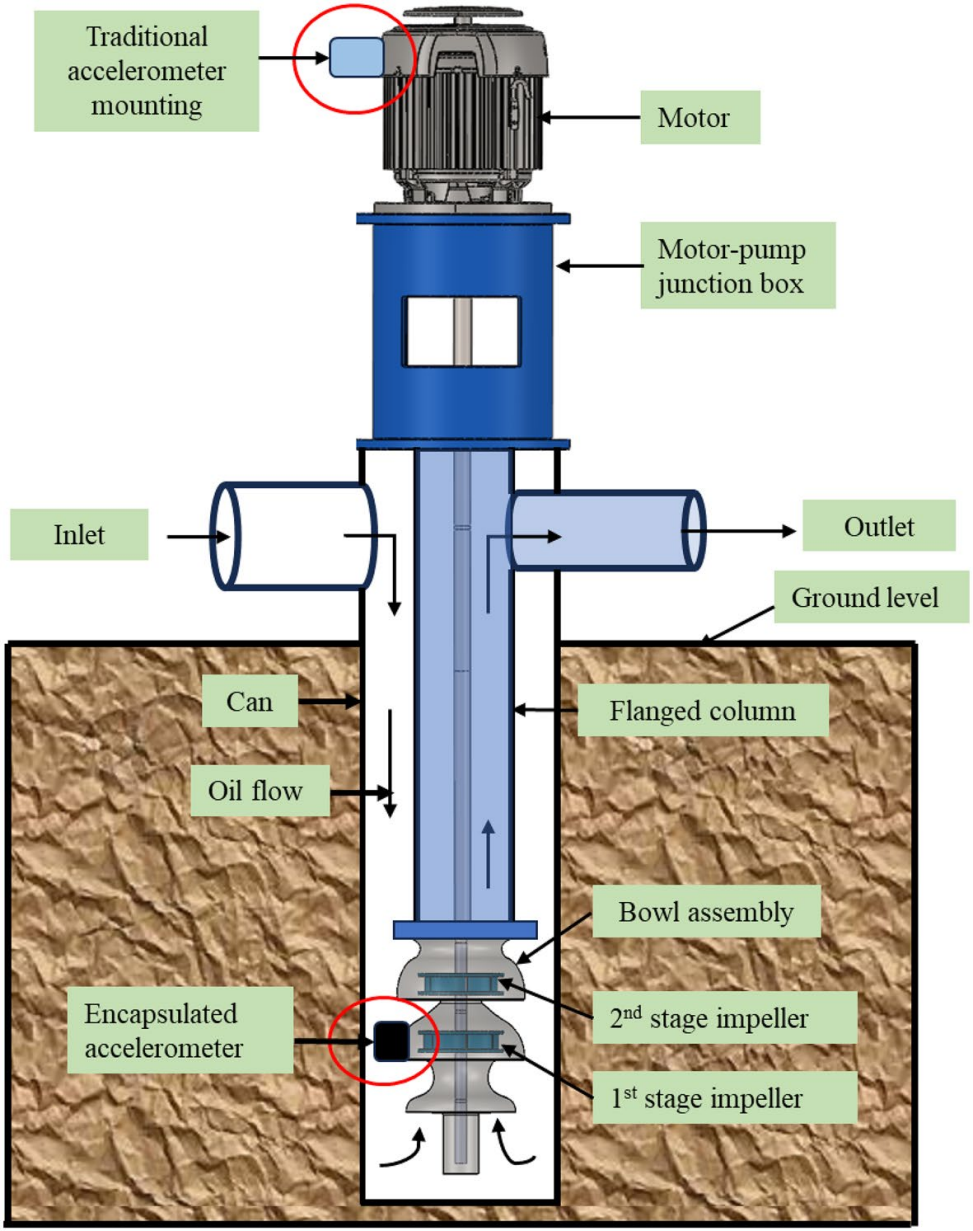
Vertical pump assembly showing accelerometers installed on the pump bowl submerged in the oil.

Figure 1 provides a visual guide to a vertically suspended closed-suction hydrocarbon pump assembly. These pumps, due to the hazardous nature of the pumped fluid, utilized a closed suction design. It features a top-mounted motor as its power source. Key components like the motor pump junction box and the inlet/outlet are located above ground. Additionally, the figure showcases a flanged pump column with a bowl assembly housing multiple impellers^9^. It also highlights the traditional placement of accelerometers on the motor and the new approach of installing them on the impeller.

**Figure 2:**
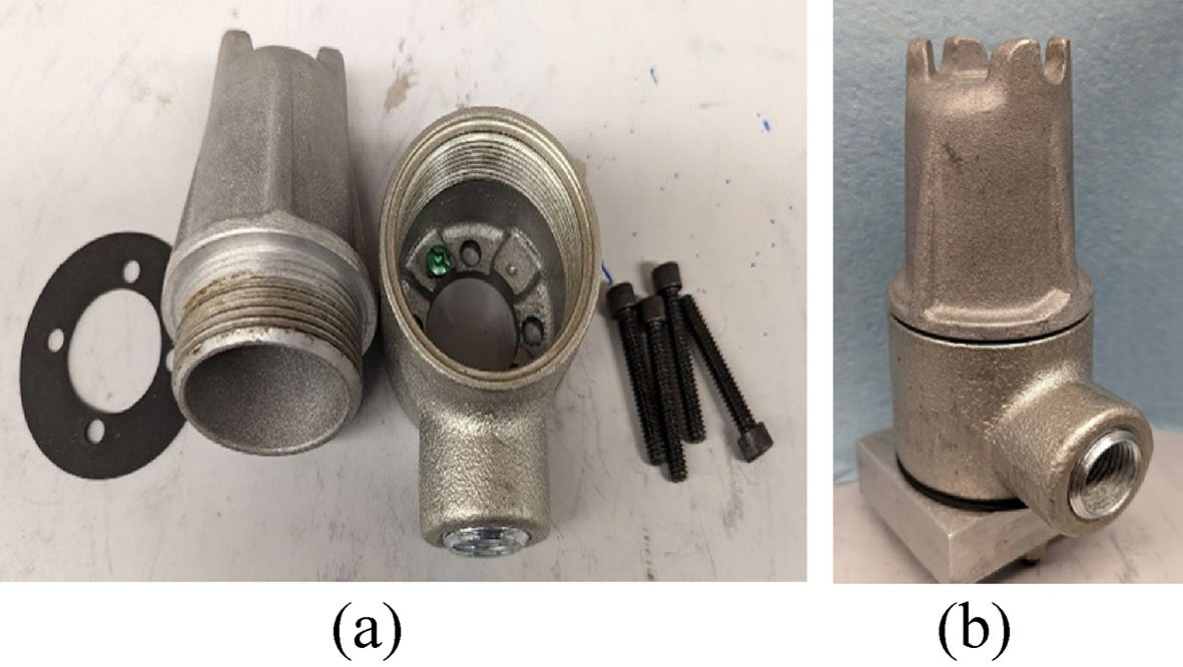
Sensor protection housing CMCP280 (**a**) Housing parts disassembly (**b**) Assembled housing on a plate.

Today, commercial accelerometers are generally rated ingress protection IP68 which means they can be submerged in water usually for 1.0-1.5 meters for up to 30 min^10,11^ however this suspects their long-term use in high-temperature hydrocarbon fluid environments. Temperature varies with geothermal gradients from 1 to 9 °C/100m of depth for downhole applications^12,13^. Considering the requirement of the project the maximum temperature limit of 150 °C was selected. In this work, commercial accelerometers will be submerged in oil at 150 °C for at least 2 years. Thus, there is a need for an additional encapsulation or enclosure for their protection. It should be chemically inert to hydrocarbon fluid, and sustain a high temperature of 150 °C. Some housings are available in the market for the protection of sensors claimed to be dust-free and watertight^14^ listed in Table 1, for instance, housing CMCP280 manufactured by STI Vibration Monitoring Inc, USA shown in Fig. 2. This housing comes with a dome cover, a mounting base with a single 3/4” NPT conduit connection, a neoprene base gasket, an O-ring gasket for the cover, mounting bolts, washers, and one 1/2” NPT reducing bushing^15^. As shown in Fig. 2, the housing features an open end that requires mounting on a plate. To make this housing suitable for submerged applications, additional accessories such as thread sealants and rubber washers must be provided. It's worth noting that there is no confirmed evidence to support the notion that the addition of external accessories can ensure their suitability for prolonged use in high-temperature hydrocarbon fluid when submerged.

**Table 1:** Commercially available housings from various vendors for the protection of accelerometers.

Vendor	Housing’s part #	Reference
Connection Technology Center (CTC), USA	MH152-1A	^16^
Connection Technology Center (CTC), USA	MH148-1A	^17^
STI Vibration Monitoring Inc, USA	CMCP280	^15^
Bently Nevada, USA	43217/37442	^14^

**Figure 3:**
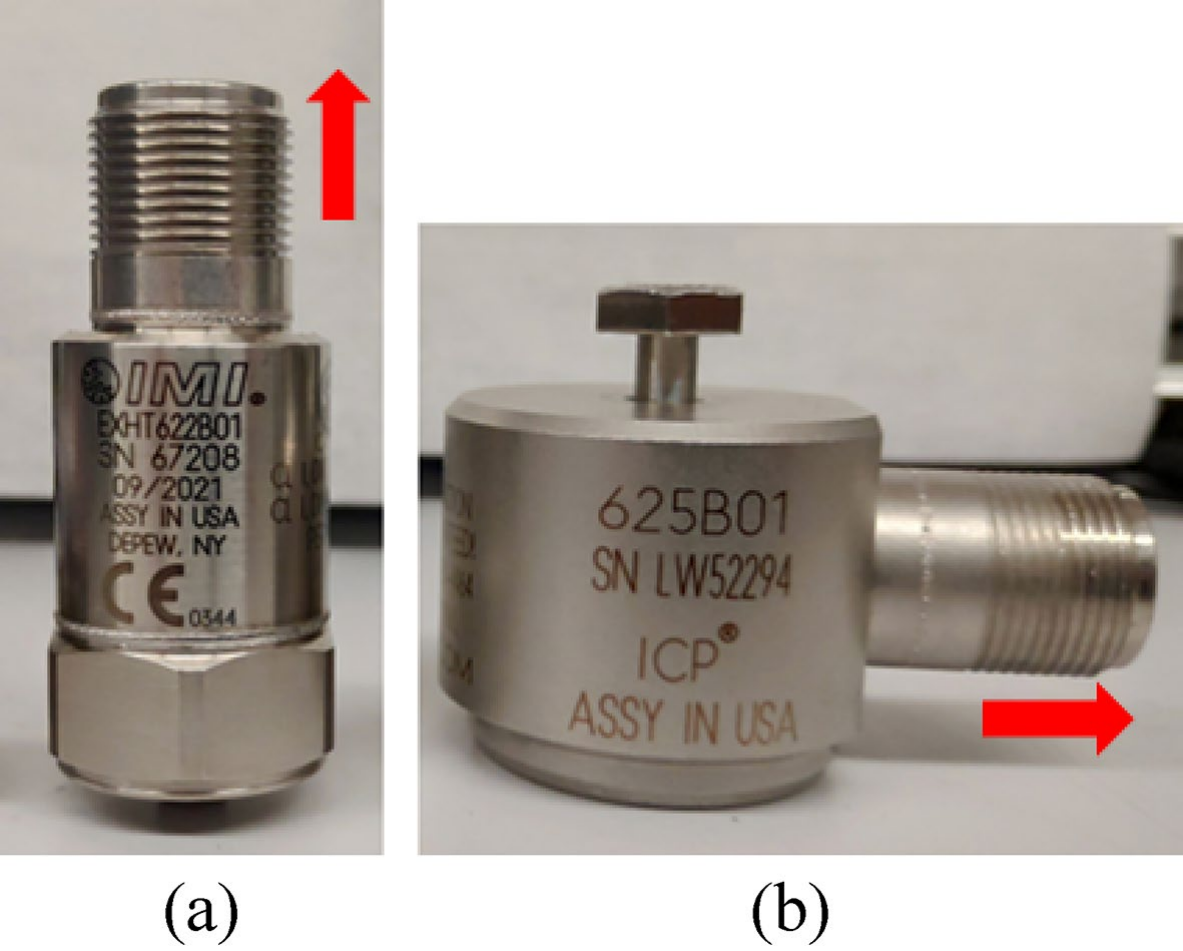
(**a**) Vertical exit accelerometer EXHT622B01 (**b**) Side exit accelerometer 625B01.

Commercial accelerometers are categorized into vertical and side exit sensors based on the orientation of the input/output wire harness connections, as depicted in Fig. 3. When connecting the input/output wire harness for the vertical exit sensor, utilize the threads located at the top of the sensor (refer to Fig. 3a). For the side exit sensor, the connection is situated on the right side (refer to Fig. 3b). Side exit sensors are mostly used in the workspace requiring low clearance and flush installation^15^. The dimensions of the commercial housing provided in Table 1 are suitable for the installation of a top exit sensor. For instance, the major diameter (seating area) of the side exit sensor 625B01 (mentioned in Table 6) is 35.1 mm, but none of the housings listed have a sufficient diameter to accommodate it. Among the listed housings, only MH148-1 is suitable for the side exit sensor. However, it lacks sealing to protect against harsh hydrocarbon fluid environments. To the best of the author’s knowledge, there is currently no sealed enclosure available in the market that is suitable for a side exit commercial sensor.

The encapsulation of accelerometers plays a crucial role in ensuring their durability in harsh hydrocarbon fluid environments. The selection of the encapsulating material is a critical decision that should be based on the operating environment, device material, and functionality. Few researchers reported packaging and encapsulation of microsystems for use in challenging high-pressure, high-temperature, and corrosive environments, such as downhole environments in the oil and gas industry. Ma et al.^18^ used stainless steel 17-4 PH metal cans with a borosilicate glass lid to package microsystems. This package was tested in high-pressure and high-salinity downhole environments within a temperature range of 75–125 °C. To enhance protection, the package was coated with thin film alumina and Parylene-C after 48 hours of exposure to API brine at 80°C. Choi et al.^19^ successfully tested autonomous sensing microsystems in extreme conditions, including temperatures and pressures up to 150 °C and 10,000 psi, along with environments containing concentrated brine, oil, and cement slurry. They utilized a hollow stainless steel 17-4 PH shell with a sapphire lid for packaging. Seren et al.^20^ and Buzi et al.^21^ developed a sensor ball designed for temperature and pressure logging in vertical wells. The sensor was encapsulated using syntactic foam material and could withstand a maximum temperature of 100 °C. Sui et al.^22^ created an Environmental Logging Microsystem (ELM) for operation in corrosive environments and at elevated pressures, with temperatures reaching up to 125 °C. The system was protected by encapsulation using 17-4PH stainless steel to guard against impact and abrasion. These studies illustrate the importance of robust packaging and encapsulation techniques for microsystems when operating in extreme and harsh conditions commonly found in the oil and gas industry. However, the housings developed by these researchers are based on their specific sensor design, which may or may not be suitable for the shape, size, and material of commercially available sensors.

While there are various materials available, it is challenging for a single material to meet all the requirements. Among researchers, epoxy resins have gained popularity as encapsulation materials for harsh environments because of their broad application range and extensive research conducted in this area. In our previous work, Lakal et.al.^8^, we reviewed epoxy resins that can withstand high-temperature oil environments, possess corrosion resistance, excellent mechanical properties, chemical and dimensional stability, and durability. Biphenyl-type epoxy resin is most popular among researchers, as it covers approximately 75% of the market share in domestic and industrial sectors^23^. Researchers like Ishii et al.^24^ have evaluated the performance of biphenyl-type epoxy in high-temperature automotive oil and oil vapor environments. According to their report, the resin, characterized by its biphenyl structure and low viscosity, offers enhanced stability and accelerated curing when combined with a phenolic resin hardener and Lewis bases. The resulting cured compound exhibits excellent mechanical and chemical properties due to its three-dimensional dense cross-linked chemical structure. Moreover, the molecular size of mineral oil is larger than the crosslink size of the epoxy resin matrix, preventing easy penetration of mineral oil into the epoxy resin matrix. The solubility parameter of epoxy resin of EMC ranged between 15-17 MPa^1/2^, while the solubility of mineral oil is estimated to be 7-8 MPa^1/2^, indicating that these materials are not soluble in each other^24^. In another study, Singh et al.^25^ investigated the effect of Epoxy/UHMWPE (ultra-high molecular weight polyethylene)/MoS_2_ composite when soaked in water, base oil, ionic liquid, and grease. They reported that the epoxy composite shows a hydrophilic nature with water, while the other liquids exhibit comparatively oleophobic behavior with the polymer. Epoxy degrades faster in a polar liquid environment than in a non-polar one. Given that oil is non-polar and epoxy is polar, the degradation is inhibited^25^. Kim et al.^26^ investigated the effect of microscale oil penetration on the mechanical and chemical properties of carbon fiber-reinforced epoxy composites. They observed thermal curing conversion of the epoxy polymer at high temperatures, through prolonged heating, leading to the formation of a densely crosslinked epoxy network that acts as an excellent oil barrier. Higher epoxy resin content creates a thick crosslinked polymer layer on the surface, which serves as a barrier to prevent oil penetration^26^. Considering its oil barrier property, we selected it as the primary encapsulation material. Moreover, numerous researchers have employed epoxy encapsulation to enable sensor usage in harsh environments across various application domains listed in Table 2.

**Table 2:** Literature on epoxy encapsulation for diverse applications in harsh environments.

Ref	Application	Harsh environment
Wnuk et.al.^27^	Fiber bragg grating strain sensors for strain monitoring: Gas and diesel engines Jet turbine blades Power plant pipeline Furnace stress measurement Stresses and crack monitoring on airplane wings Rocket boosters and fuselages	Strain (+ / − 1000µe) and temperature (− 20 to + 120 °C)
Linz et.al.^28^	Textiles for wearable electronics	Washing at 40 °C and 60 °C, perspiration, temperature cycles, humidity, Flex, Crumpling and Stretch, and Abrasion
Birkelund et.al.^29^	MEMS-based multi-sensors for fisheries research	Pressure from 1 to 30 bars and at temperatures ranging from -20 °C to 25 °C for 2 years in seawater
Qin et.al.^30^	Embedded Wireless accelerometer for condition monitoring of helicopter planetary gearbox	Complex transmission systems of the helicopter
Ma et.al.^31^	Fiber Bragg Grating temperature sensor with high-pressure resistance for downhole	Temperature range of 0 °C to 70 °C and pressure range of 0–20 MPa
Boeser et.al.^32^	Implantable electronic devices in the human body	Humid conditions inside the human body
Stosur et al.^33^	Power electronics components used in pressure vessels for the navy	High pressure 310 Bar
Spanier et.al.^34^	Electronics for geothermal application	Submerging sensors for 1000 h in a water bath at 100 °C and in Glycerol at 150 °C

In addition to epoxy, few researchers have reported the utilization of resorcinol-based phthalonitrile (rPN) as an encapsulation material for high-temperature applications reaching up to 300 °C^35,36^. While there have been numerous studies on encapsulating sensors across various applications, our focus in this work is to contribute to the field of downhole applications.

In commercial accelerometers, power, and signal transmission are typically accomplished through wire harness connections. Therefore, encapsulating the wire harness in harsh conditions is critical. Considering the flexibility of the wire harness, we need encapsulation, which is flexible, resistant to high oil temperature, chemically inert, and durable. Fluoroelastomer (FKM), first developed by Chemours under the brand name Viton and defined by the ASTM International standard D1418, meets these requirements^37,38^. FKM is widely used in aerospace, military, oil and gas, and chemical processing industries, specifically in applications with extreme temperatures, pressures, and chemical surroundings. It exhibits exceptional resistance to ozone, oils, aging oxidizers, aromatic and aliphatic hydrocarbons, and a variety of chemicals, and demonstrates phenomenal performance in a highly corrosive and hot environment^38,39^. Fluorine is the highest electronegative among all halogens which enables them to form significantly stronger bonds compared to other elastomers, which makes it less prone to degradation^40^. This characteristic makes FKM less prone to degradation. It can retain its properties even at temperatures up to 200°C and exhibits a tensile strength of 11 MPa, along with an elongation at fracture of about 200%^40^. Numerous researchers have used FKM for its excellent sustainability in harsh environments. For instance, Vellaluru et al. encapsulated an autonomous microsystem with FKM for application in harsh fluid environments such as diesel fuel, brine, and hydrogen sulfide (H_2_S)^41^. Sharma et al.^40^ demonstrated the use of FKM-based microfluidics that effectively handle oil and gases without experiencing leakages or swelling in pumps, mixers, and hydraulic systems. Recently, FKM has also been used as an encapsulant in flexible electronics^40^. Monshi et al. used FKM to restrict the moisture interaction and permeation in electronics embedded in textiles^42^. Seo et al. presented a reliable, hysteresis-free, and bias-stable performance of single-walled carbon nanotube (SWCNT) thin-film transistors (SWCNT-TFTs) through FKM encapsulation^43^. Takahashi et al. demonstrated a microcantilever-based tactile sensor ingrained in FKM/PDMS to protect it from external chemical and physical repercussions^44^. Considering the maximum operating temperature and environmental resistance, we compared several sealing rubbers listed in Table 3, and FKM emerged as our preferred choice as the second encapsulation material.

**Table 3:** Classification of various rubbers based on operating temperature and environmental resistance.

Properties	Fluoroelastomer (FKM)	Perflouro elastomer (FFKM)	Ethylene propylene diene monomer rubber (EPDM)	Buna-N, Nitrile rubber (NBR)	Neoprene	Silicone Rubber
Maximum temperature for continuous use (°C)	260	325	177	120	120	204.44
Environmental Resistance						
Hydrocarbon Oils/ greases/Fuel/Solvents	Excellent	Excellent	Poor	Fair to good	Poor to fair	Fair
Ozone	Outstanding	Excellent	Outstanding	Poor	Very good	Excellent
Oxidation	Outstanding	–	Excellent	Fair-good	Very good	Excellent
Weathering (wearing)	Excellent	Excellent	Outstanding	Good	Very good	Excellent
Water	Good	Excellent	Excellent	Excellent	Good	Excellent
Radiation	Fair-good	–	Good	Fair-good	Good	Good
Reference	^45,46^	^47,48^	^49^	^50,51^	^52^	^53–55^

We used a multilayer structure for encapsulating commercial accelerometers and accessed their performance in a high-temperature hydrocarbon fluid environment. Epoxy was used as the primary encapsulation layer, protecting the sensor from a high-temperature hydrocarbon fluid environment. FKM served multiple functions as the second encapsulation layer. It helped mitigate the effect of thermal expansion between the steel sensor body and the epoxy resin, acted as a heat insulator, and provided an additional protection layer against oil leaks. Furthermore, we employed an FKM hose to safeguard the sensor wire harness from the harsh hydrocarbon fluid environment. We evaluated epoxy and FKM by subjecting them to high-temperature hydrocarbon fluid at 150 °C for more than a year. Through material characterization such as mass variation analysis, microscopic examination using a confocal microscope, and molecular structure assessment using FTIR, we measured the changes in encapsulation material that occurred over a year. Apart from hydrocarbon fluid, we also tested the encapsulation materials in the API brine solution for half a year. A computer-aided design (CAD) design was developed, considering the geometry of the sensors, for encapsulating the side exit and vertical exit accelerometers. Finite element method (FEM) simulations were conducted using the material properties to analyze stress and strain distribution resulting from high temperatures. Further, the housing was cast using a 3D-printed mold, and the encapsulated accelerometer’s performance was evaluated for vibration sensing. Finally, we performed oil leak tests to compare the performance of the commercial housing and the housing developed in this study.

The purpose of this research is to assess whether epoxy and fluoroelastomers can be used as effective materials for enclosing sensors that need to be immersed in high-temperature hydrocarbon fluids for extended periods. We conducted extensive tests on the encapsulation material, submerging it in high-temperature hydrocarbon fluid at 150 °C for over a year (more than 10,000 hours) and for six months in brine. We assessed the material's mass variation, microscopic imaging, and FTIR spectroscopy and found that it had minimal degradation. When we packaged accelerometers in the encapsulation, they were able to successfully detect vibrations with only minimal changes in amplitude. We compared our encapsulation with commercially available options and found that the latter had traces of oil within 24 h, while our housing had no leaks. Notably, this study represents the first instance of a sealed packaging enclosure being developed and tested using epoxy resin and FKM for a side exit commercial sensor in a high-temperature hydrocarbon fluid environment.

## Experimental details

### Materials

We identified two epoxies that satisfied our requirements, showing excellent resistance to hydrocarbon fluids and a capacity to endure high temperatures. These epoxies were bought from 1) PC Fahrenheit from Protective Coating Company, USA, and 2) LOCTITE STYCAST 2762FT from Henkel Corporation, USA. Epoxy#1 PC-Fahrenheit, has a temperature resistance of up to 500°F(260 °C) and resistance to various substances, including hydrocarbons, ketones, alcohol, esters, aqueous salt solutions, halocarbons, dilute acids, and bases^56^. The main chemical constituents of this epoxy are outlined in Table 4.

**Table 4:** Chemical components of PC-Fahrenheit epoxy^56^.

Chemicals components	Weight-%
Bisphenol A—Epichlorohydrin polymer	10–30
2,4,6-Tri(dimethylaminomethyl)phenol	1–5
Crystalline silica	0.1–1.0

Epoxy #2, LOCTITE STYCAST 2762FT, possesses desirable characteristics, such as high thermal conductivity, high-temperature resistance of up to 230 °C, and excellent chemical resistance to hydrocarbon fluids^57^. Its main chemical constituents are mentioned in Table 5*.*

**Table 5:** Chemical composition of LOCTITE STYCAST 2762FT^57^.

Chemicals composition	Weight-%
Aluminum oxide—nonfibrous form	60–80
Epichlorohydrin-4,4'-isopropylidene diphenol resin	10–30
Bisphenol-F Epichlorohydrin resin	1–5
Butyl glycidyl ether	0.1–1
Carbon black	0.1–1

Accelerometer models EXHT622B01 (vertical exit) and 625B01 (side exit) from PCB Electronics, USA were used in this work^58,59^. Table 6 outlines the crucial characteristics of the two sensors, while Fig. 3 displays their corresponding images.

**Table 6:** Specification of the accelerometer^58,59^.

Model	EXHT622B01	625B01
Operating temperature range	( −)54 °C to ( +)163 °C	( −)54 °C to ( +)121 °C
Housing material	Stainless Steel	Stainless Steel
Enclosure rating	IP68	IP68
Size	Height = 52.3 mm Diameter = 22.10 mm Hex = 22 mm	Diameter = 35.1 mm Height = 28.7 mm
Frequency range	0.2 to 15,000 Hz	0.2 to 10,500 Hz

The FKM sheet used in this study was supplied by Grainger, USA, and it has a specified operating temperature range of − 24 °C to 204 °C. We purchased Viton^®^ FKM opaque tubing from McMaster-Carr, USA. Further, the hydraulic oil (MAG 1 AW ISO 46 hydraulic oil) was sourced from Grainger, USA. For formulating the brine solution, we purchased chemicals sodium chloride and calcium chloride, from Sigma Aldrich, USA.

### Preparation of the epoxy specimen

Epoxy samples for testing in hydrocarbon fluid were prepared using an in-house 3D-printed mold. The process flow was set up by first making cubes of the silicon release liner and pouring liquid epoxy. Epoxy 2762FT was cured at 3 h at 125 °C and 3 h at 175 °C and epoxy PC Fahrenheit was cured for 60 min at room temperature^56,57^. Epoxy cubes with a similar shape and size were obtained after curing. They were submerged in an insulated in-house oil bath at 150°C. The entire process flow is shown in Fig. S1 Supplementary information.

### Characterization

The primary aim of this characterization process is divided into two parts: firstly, to establish a method for examining potential interactions and penetration between hydraulic oil and epoxy, and secondly, to ensure that the encapsulation does not compromise the accelerometer's performance. Drawing inspiration from the methodologies employed by various researchers, our approach combines elements from studies conducted by Ishii et al.^24^, Kim et al.^26^, Singh et al.^25^, Rocha et al.^60^ for the first objective, and by Jiang et al.^61^ and Fort et al.^1^ for the second objective.

Consequently, the characterization process is structured into two distinct phases:

1. Encapsulation material characterization: This phase focuses on assessing the properties of the encapsulation material and its compatibility with hydraulic oil.
2. Accelerometer vibration measurement: In the second phase, the objective is to gauge the accelerometer's performance with the encapsulation in place.

**Figure 4:**
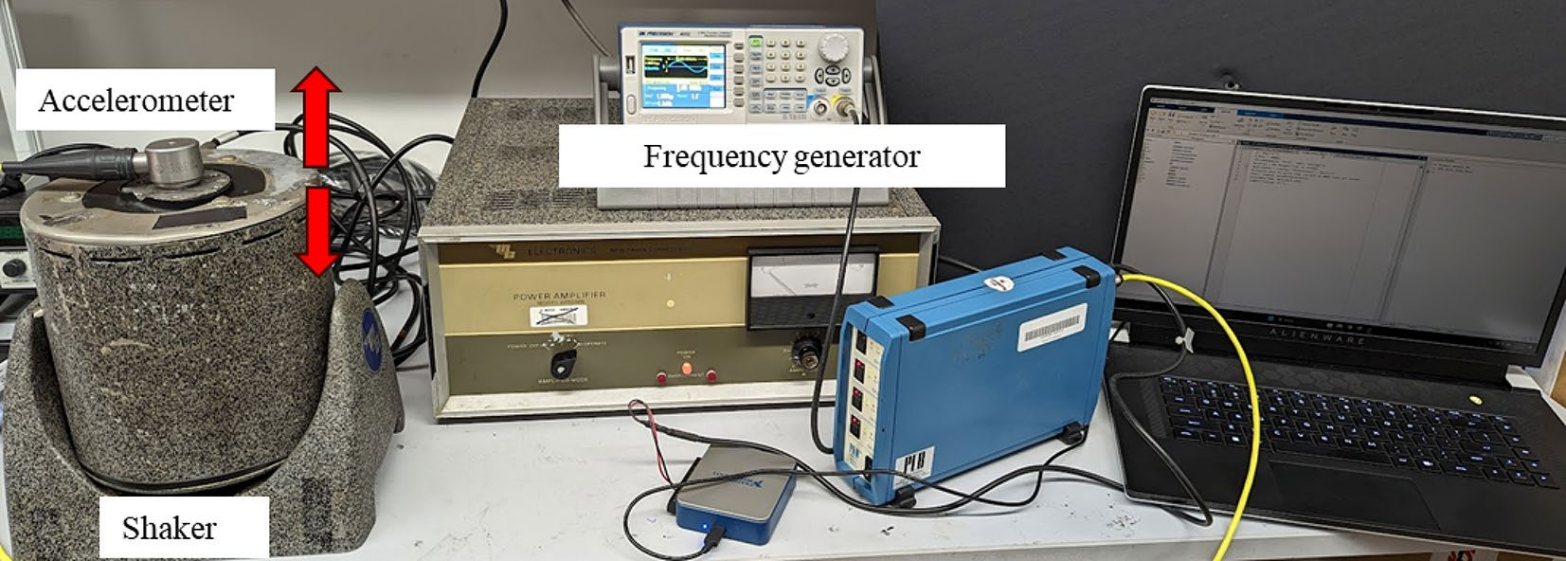
Vibration measurement setup showing vibration motion of the shaker (red arrow), frequency generator, and accelerometer.

In the first part, the epoxy and FKM samples were submerged in hydraulic oil and their weight was measured every week using a Mettler Toledo XS1203S weighing scale. To analyze any changes in the chemical composition, FTIR spectra were obtained for both the control sample and the treated samples using a PerkinElmer—Frontier MIR/FIR instrument. Confocal microscopy was performed using the CrestV2 microscope with 2xTIRF to examine any microscopic changes in the encapsulation materials. For the second part, a vibration measurement setup was utilized, as depicted in Fig. 4. The setup consists of a vibration shaker table, a frequency generator, and a vibration data acquisition system. Accelerometers were tested before and after encapsulation to evaluate the change in vibration-sensing capabilities.

## Results and discussions

### Multilayer encapsulation structure design

**Figure 5:**
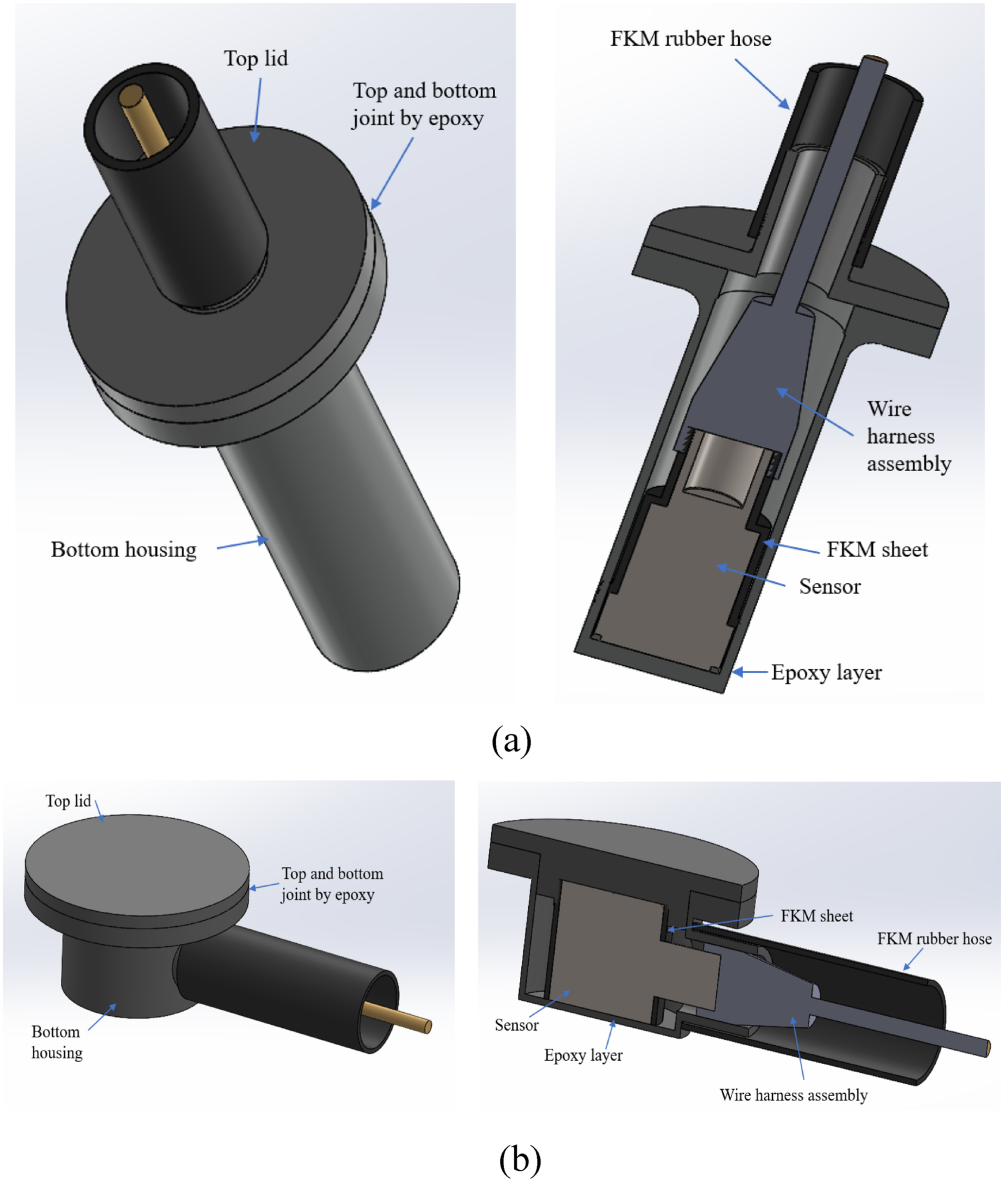
Multilayer encapsulation structure for (**a**) Vertical exit sensor (**b**) Side exit sensor.

Figure 5 illustrates the design of a multilayer encapsulation structure that was developed to safeguard the commercial sensor from the harsh hydrocarbon fluid environment.

A CAD model of the encapsulated sensor was created using Solidworks software. The outer appearance of the model showcases the first layer of epoxy encapsulation, followed by the FKM layer. The casing was divided into two parts: the top lid and the bottom housing. The dimensions of the bottom housing and top lid were defined to accommodate the entire sensor along with the connector assembly. The sensor was first inserted into the bottom housing with the wire harness assembly and the top lid was glued to the bottom housing using the same epoxy.

To ensure the safety of the designed structure, the FEA (Finite element analysis) was performed to determine the stress generated between the interfaces, considering different thermal coefficients of expansion of the materials in this assembly i.e., stainless steel of the sensor, FKM, and epoxy. The FEA simulation was performed by applying the maximum temperature of 150 °C. Since epoxy is brittle after curing and is exposed to high-temperature hydrocarbon fluid, we determined the factor of safety considering the ultimate compression strength of the epoxy. The ultimate compression strength of the epoxy was measured using the Universal testing machine Instron 3345 and found to be 158 MPa (see Fig. S2 supplementary information). The average stress generated in the epoxy at the intersection zone was found to be 13.5 MPa shown in Fig. S3 supplementary information which is less than 158 MPa. The Mohr–Coulomb factor of safety criteria was considered in this case in Fig. S4 supplementary information found 2.8 (greater than 1) which justifies safe design.

A rubber sheet was placed between the contact surfaces of the steel and the epoxy to prevent the generation of cracks and failure of the encapsulation due to the thermal strain caused by the difference in thermal expansion coefficients between epoxy (~ 3.8 × 10^–5^/°C)^57^ and the 316 stainless steel (~ 1.6 × 10^–5^/°C)^62^. The strain distribution analysis, as depicted in Fig. S5 supplementary information, highlights a key observation. When FKM is introduced, the thermal strain within the model doesn't propagate to the epoxy and steel body. In contrast, the model lacking FKM shows the transfer of thermal strain to the epoxy outer shell. This finding underscores the efficacy of the FKM layer in mitigating thermal strain transfer.

**Figure 6:**
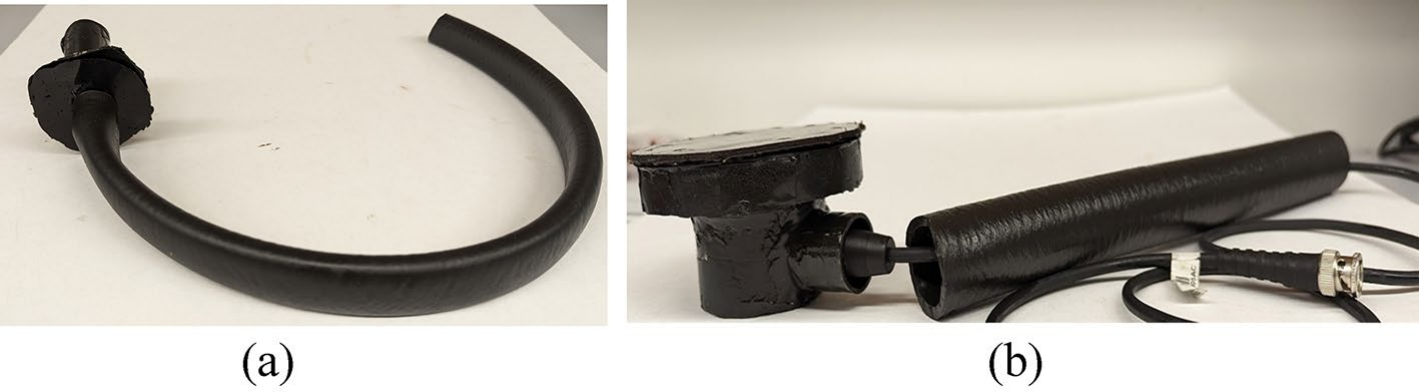
Encapsulated assembly for (**a**) vertical exit sensor, and (**b**) side exit sensor.

Epoxy housings were fabricated by casting them within a custom-made 3D-printed mold, tailored specifically for accommodating both side exit and vertical exit sensors, Fig. 6 showcases the final fabricated parts of the assembly.

Further characterization of the encapsulation material involves conducting individual tests on epoxy and FKM in a high-temperature hydrocarbon fluid environment. The results of the tests are discussed in the consequent sections.

### Epoxy mass variation test

**Figure 7:**
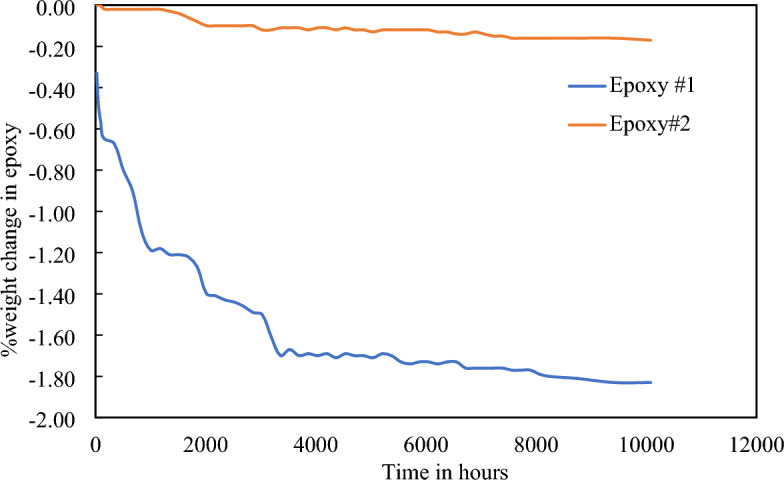
Change in weight of epoxies submerged in hydraulic oil at 150 °C.

Measurement of weight aids in quantitatively tracking the degradation process over time. It helps researchers to understand the behavior of the material, extent of degradation, and rate of degradation in the environment they are subject to^60^. In this work, Epoxy is the prime material for encapsulation, and to assess its degradation when submerged in the hydrocarbon fluid at 150 °C, a mass variation test was conducted. The weight of the epoxy samples was recorded before submerging them in the oil and measured every week for over a year (> 10,000 h.)*.* Figure 7 shows the percent change in the weight of the epoxy in the hydraulic oil. Percentage change in weight was calculated using the Eq. (1)^60^:

1 $$\mathrm{\%weight\, change}= \frac{\mathrm{Final\, weight}-\mathrm{Initial\, weight}}{\mathrm{Initial \,weight}}\times 100$$

Table 7 provides a concise summary of the percentage weight change in epoxy.

**Table 7:** Summary of percent weight change in epoxy in hydraulic oil at 150 °C.

Time (hrs.)	% weight change in epoxy in hydraulic oil at 150 °C	
	Epoxy #1: PC Fahrenheit Epoxy Putty	Epoxy #2: LOCTITE STYCAST 2762FT
10,080	− 1.83	− 0.17

From Fig. 7 it is evident that the weight of epoxy #2 is stable throughout the test. For epoxy#1, weight decreases by more than 1% as compared to epoxy#2. The reason can be attributed to the difference in the content of both epoxies. Epoxy #1 is formulated with Bisphenol-A (Bis-A), which is a general-purpose epoxy resin, while Epoxy #2 incorporates Bisphenol-F (Bis-F), a tightly cross-linked epoxy resin with superior chemical resistance properties^63,64^. Furthermore, Bisphenol-F epoxy resins exhibit low viscosity, facilitating ease of moldability and workability^65^. The epoxy-oil weight experiment revealed an approximate < 1% change in weight for epoxy#2 over a year. Consequently, for subsequent characterization, the focus was solely on epoxy#2.

### Microscopic characterization of oil epoxy interaction

**Figure 8:**
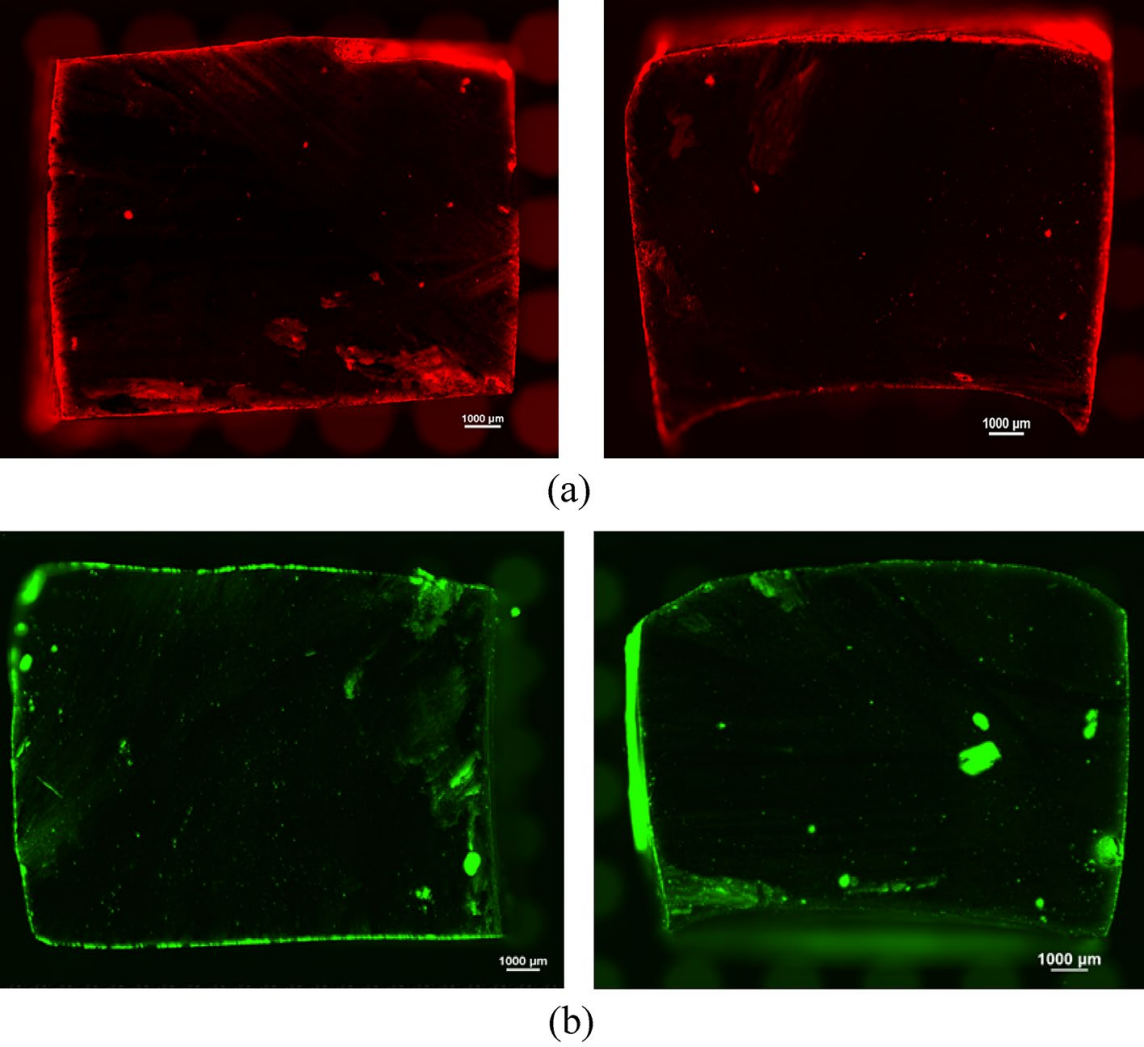
Cut section of the two epoxy samples submerged in the oil at 150°C, showing no traces of oil inside the cut section. (**a**) Sample 1 and Sample 2 submerged in red dye and oil mixture (**b**) Sample 1 and Sample 2 submerged in green dye and oil mixture.

Our goal of characterization was to examine the penetration or any effect of hydraulic oil on epoxy during their interaction. Since hydrocarbons are present in both oil and epoxy, it is difficult to distinguish between them. To identify oil traces, we introduced a fluorescent color dye label by mixing the hydraulic oil with red and green, fluorescent color dye and examined them under a confocal microscope. We used two florescent dies (1) Biotium Nile Red from Fisher Scientific, USA, and (2) Oil Glo green ultra-UV fluorescent dye from Spectroline, USA, to prepare the solution. Fig. S6 supplementary information shows the Nile red and Oil Glo solution. Epoxy samples were submerged in this mixture of oil and color die. These samples were cut in half and checked under a confocal microscope for fluorescent traces. Figure 8 shows the cut sections of the two epoxy samples submerged in the oil at 150 °C. Although we observed fluorescent traces on the edges of the material, we did not detect any traces of the fluorescent material inside the cut section.

Some isolated spots of fluorescence were observed inside the cut section, which could have resulted from the cutting operation. Thus, based on these microscopic images we can confirm that oil did not penetrate epoxy. To further validate this at a molecular level, we performed spectroscopic analysis using FTIR, as described in the following section.

### FTIR spectroscopy characterization of oil and epoxy interaction

**Figure 9:**
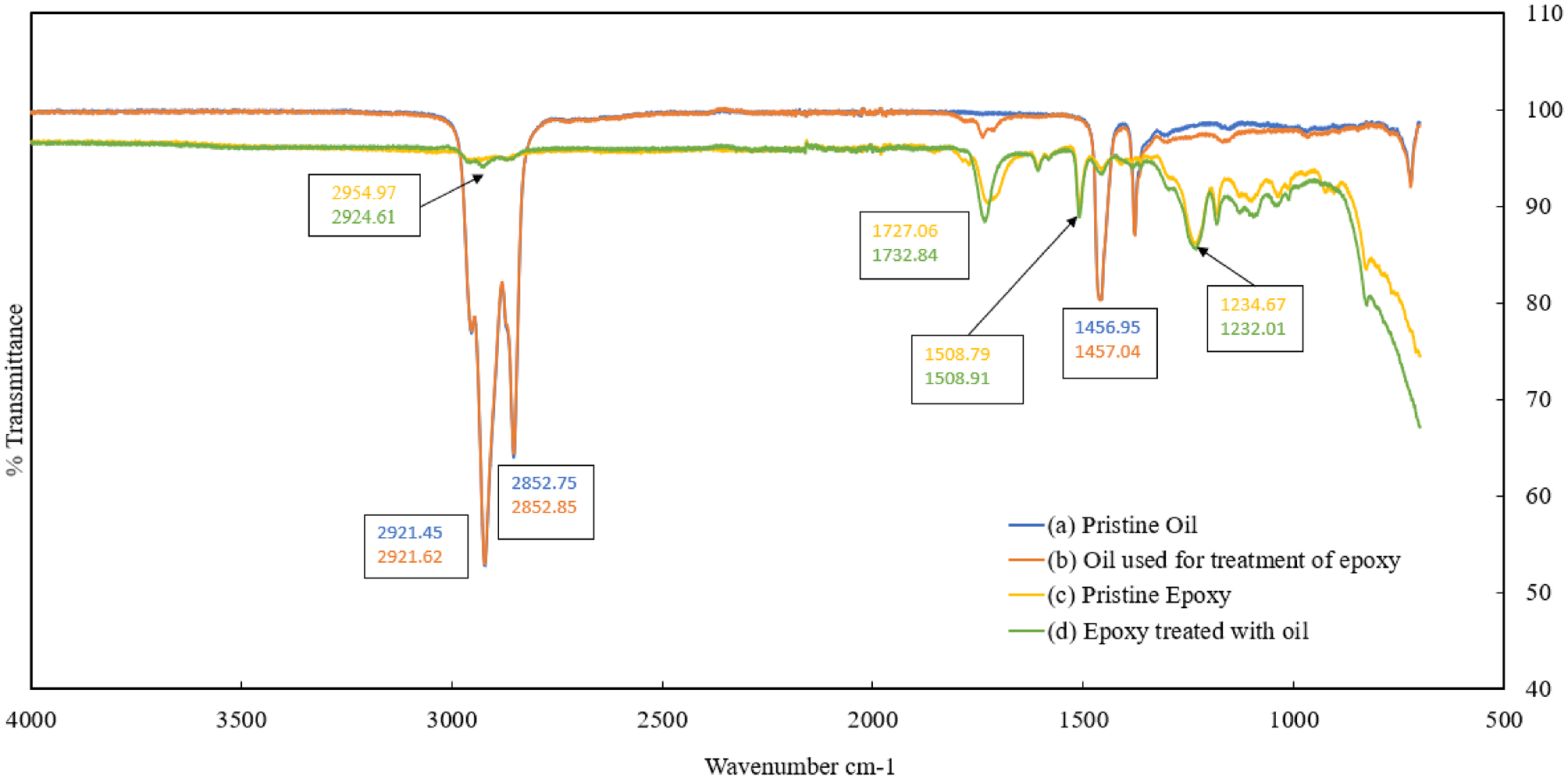
FTIR spectra of pristine and treated epoxy and oil show negligible changes in the peaks after the interaction.

FTIR spectroscopy was conducted to assess molecular level changes in the epoxy caused by the presence of oil. FTIR plots were generated for the epoxy submerged in the hydraulic oil over a year. Four FTIR spectra were analyzed to evaluate the epoxy-oil interaction similar to the study reported by Kim et al.^26^ The FTIR spectra included (a) pristine oil, (b) oil used for the treatment of the epoxy, (c) pristine epoxy (i.e., epoxy samples that had not been submerged in the oil or control sample), and (d) epoxy samples submerged in the oil. Fig. 9 shows the FTIR spectra of all four categories of the samples.

From Fig. 9, it is evident that spectra of the pristine oil (a) and the oil used for the treatment of epoxy (b) exhibit similar peaks, indicating their consistency. Similarly, the peaks in the spectra of pristine epoxy (c) and the epoxy treated with oil (d) are comparable. None of the peaks from the spectra of the oil are observable in the spectra of the epoxy and vice versa.

For oil, double C-H stretching bonds are typically observed between 2800–3000 cm^−1^, and single C-H bonds are found around 1455 cm^−1^^26^. Similarly for pristine epoxy C-H stretching bonds are found between 2920 cm^−1^ and 2850 cm^−1^. Stretching of carbonyl groups is found at a wavenumber of 1728 cm^−1^ and 1747 cm^−1^, and stretching of the benzene ring is found in 1610–1510 cm^−1^ and 1458 cm^−1^. 1230–1250 cm^−1^ and 1025 cm^−1^ are the stretching mode for aromatic ether^60^. Based on the spectra, it is evident that none of the wavenumbers corresponding to these bonds are unaltered before and after treatment, this implies oil and epoxy interaction, in this case, was insignificant.

### FKM mass variation test in oil

We used FKM hose for connecting wires, and secondary sealing. This FKM will also encounter high-temperature hydrocarbon fluid. Hence, it is essential to study the performance of FKMs in high-temperature hydrocarbon fluids. While we have reported on the FKM performance in high-temperature hydrocarbon fluid in our previous work (Wankhede et al.^30,66^), we reevaluate it in this study for a longer duration.

The degradation of FKM submerged in oil was evaluated by a mass variation test. We compared several rubbers used for sealing, as described in Table 3 by considering their maximum operating temperature and environmental resistance. Silicone rubber, FKM, and FFKM were reported to have a similar maximum operating temperature of 200 °C. However, due to its high cost, FFKM was disregarded. Therefore, FKM and silicone rubber were submerged at an equal temperature of 150 °C for the same duration.

**Figure 10:**
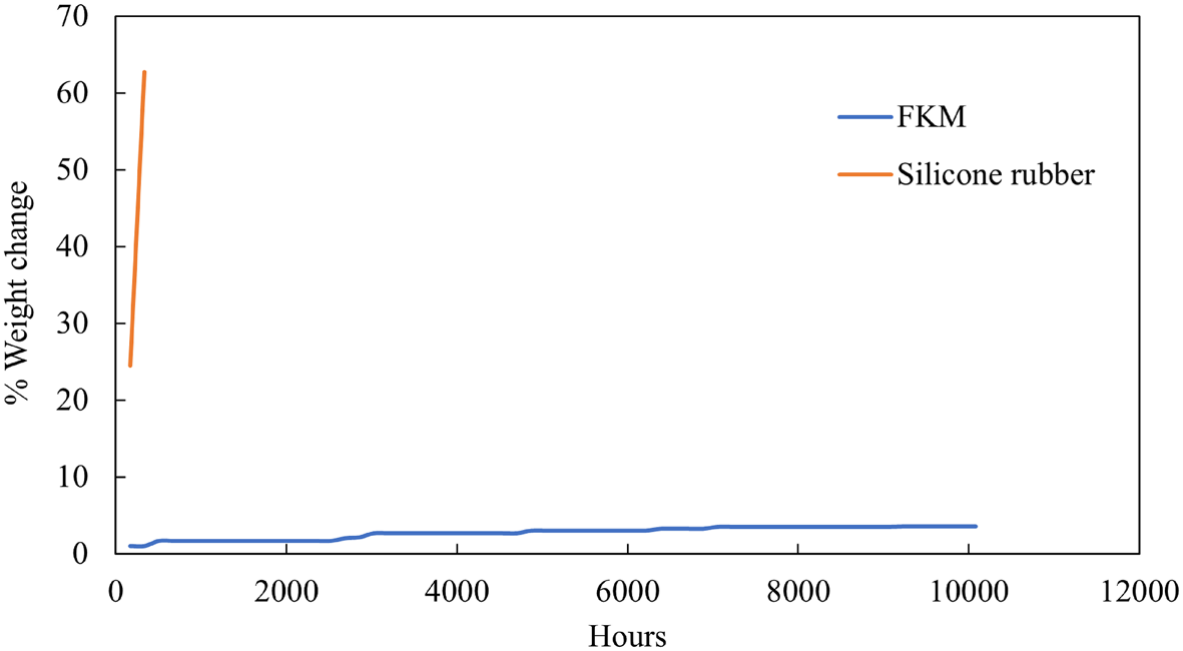
Percent weight change of FKM and silicone rubber submerged in oil at 150 °C.

**Figure 11:**
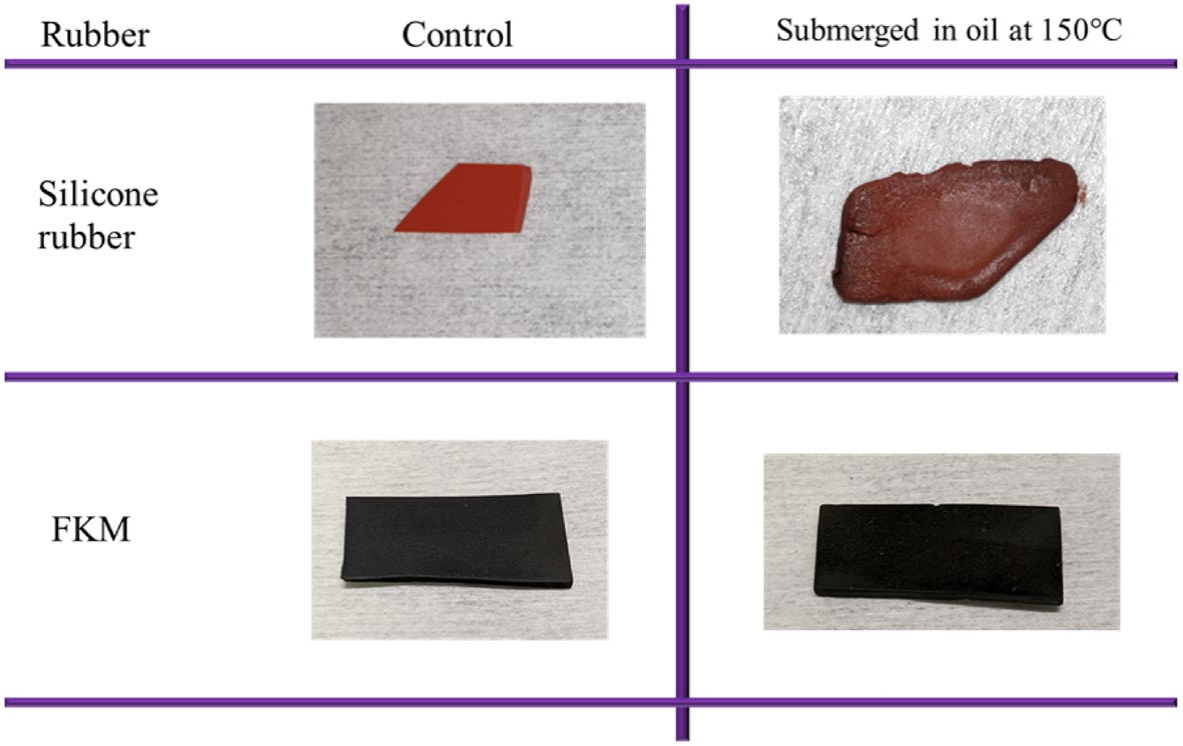
Physical appearance of silicone rubber and FKM before and after the test.

The weight of FKM and silicone rubbers were measured before submerging them in the oil and subsequent measurements were taken weekly. Significant changes in the physical appearance and weight of the silicone rubber were observed, leading us to terminate the test within 2 weeks. However, the evaluation of FKM continued testing FKM for over a year. The percent weight change for silicone rubber and FKM is illustrated in Fig. 10*.* Table 8 shows the percent change in weight of the FKM and silicone rubber in the hydraulic oil. The percentage change in weight was calculated using Eq. (1). Figure 11 shows the physical appearance of FKM and silicone rubber before and after the tests. Silicone rubber showed a significant change in weight because of swelling and developed cracks due to its incompatibility with hydrocarbon fluids^67,68^. On the other hand, FKM showed no change in physical appearance or weight. This can be attributed to the high electronegative fluorine atoms in the FKM, which enhances its resistance degradation when exposed to high-temperature hydrocarbon fluids.

**Table 8:** Change in the weight of FKM.

Substrate	Duration (hours)	% Weight change
Silicone rubber	336	62.69
FKM	10,080	3.54

### Microscopic characterization of oil FKM interaction

**Figure 12:**
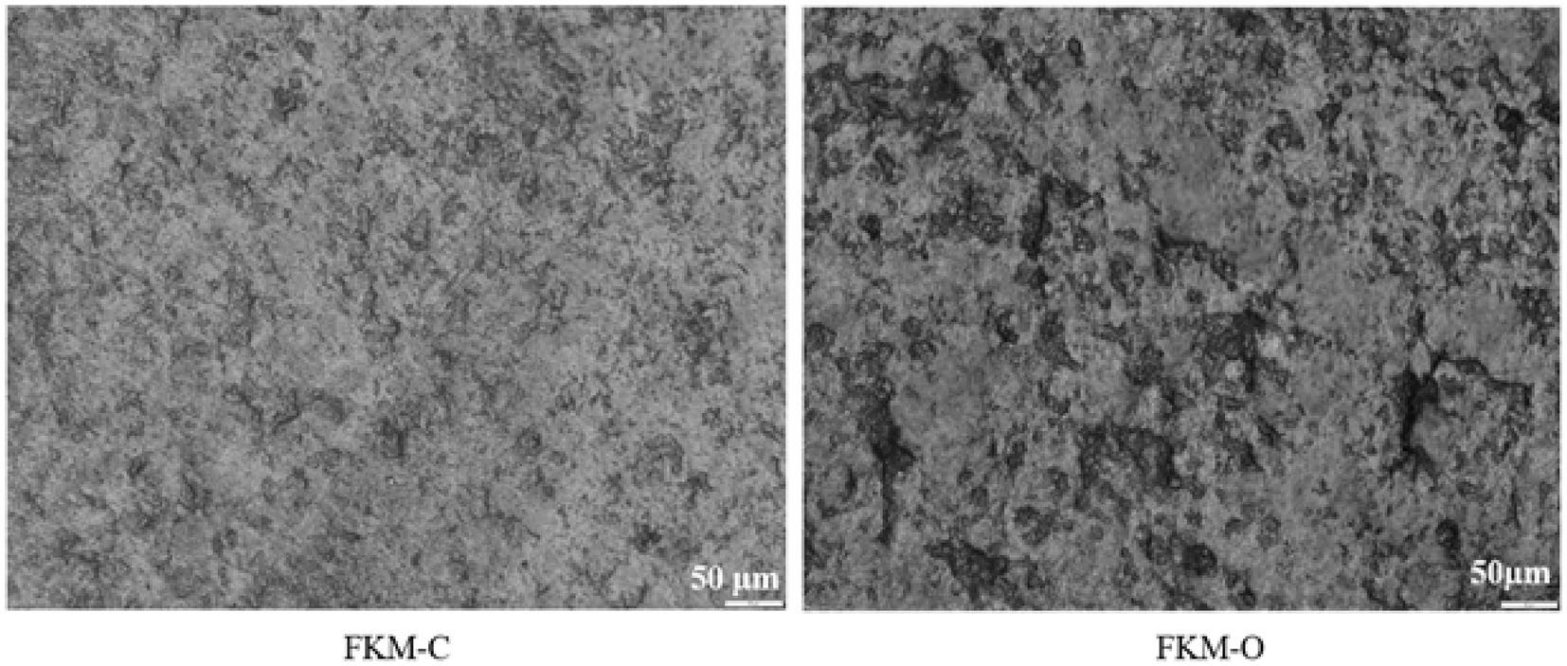
Microscopic images of the FKM-C and FKM-O.

The effect of high-temperature oil on the microstructural changes of FKM was examined through microscopic images. To simplify identification and understanding, we labeled the samples treated with oil as FKM-O (FKM submerged in oil at 150 °C for 10,080 h), and the untreated control samples as FKM-C. Figure 12 shows the microscopic images of the FKM-O and FKM-C. Observing the images, it is evident that the surface of FKM-C samples appears to be less rough than FKM-O. To quantitively analyze this difference, surface roughness (R_a_) measurements were conducted on both samples using a 3D optical profilometer (Zygo, Nexview™). The surface roughness R_a_ in µm was measured at five locations on each FKM-O and FKM-C sample, and the results are presented in Table 9.

**Table 9:** Surface roughness R_a_ (µm) of the FKM-C and FKM-O.

Position	FKM-C	FKM-O
1	0.902	0.998
2	0.967	1.104
3	1.102	1.018
4	0.905	0.998
5	0.902	0.998
Average	0.945	0.979

From Table 9, it is evident that FKM-O exhibits marginally higher surface roughness compared to FKM-C. This suggests a marginal degradation of FKM after being treated in oil at 150 °C for over a year. However, no significant cracks, particle agglomeration, or voids were observed in FKM-O. This indicates FKM’s ability to withstand the high-temperature hydrocarbon fluid environment without experiencing severe structural damage.

### FTIR spectroscopy characterization of FKM and oil interaction

FTIR spectroscopy was conducted to evaluate the molecular changes of FKM in a harsh environment. FTIR spectra for FKM-O and FKM-C have been graphed, following the methodology reported by Kim et al.^26^

**Figure 13:**
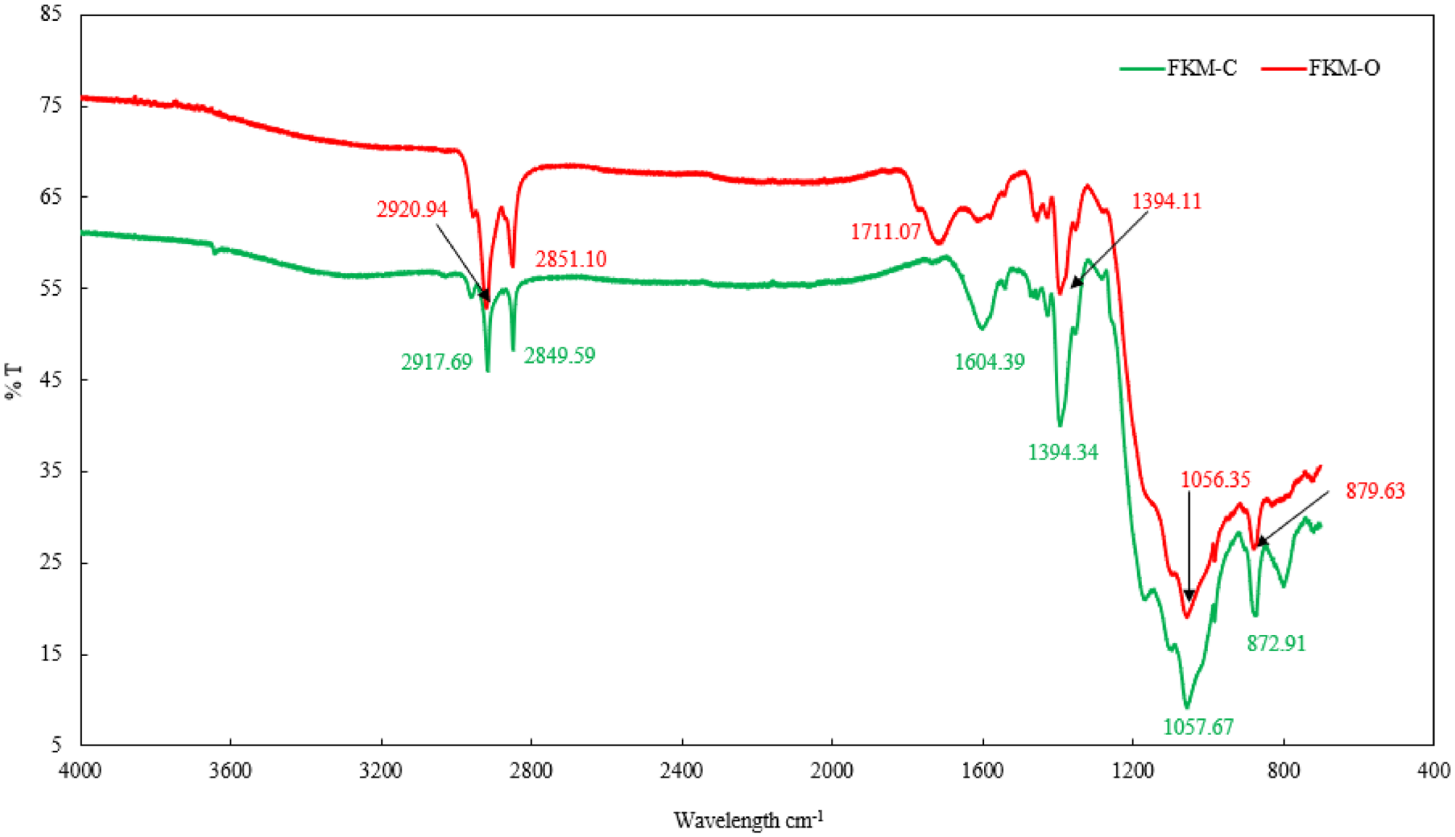
FTIR spectra of FKM-O and FKM-C show negligible changes in the peaks.

Figure 13 shows the FTIR spectra of FKM-O and FKM-C. The Type 1 FKM structure is characterized by CF_3_, CF, CF_2,_ and CH_2_ bonds^69^. The peak at 1057.67 cm^−1^, corresponding to the strongest bond CF_2_, remained intact in the FKM-O sample at 1056.35 cm^−1^ even after 1 year of treatment. The characteristic peaks of CF at 1394.34 cm^−1^ and CF3 at 872.91 cm^−1^ exhibit minimal changes to 1394.11 cm^−1^ and 879.63 cm^−1^ respectively. The CH stretching peaks at 2917.69 cm^−1^ and 2849.59 cm^−1^ show negligible changes to 2920.94 cm^−1^ and 2851.10 cm^−1^ respectively. However, the C = C stretching peaks show a slight shift from 1604.39 cm^−1^ to 1711.07 cm^−1^.

The shift of the peak to a higher wavenumber implies a decrease in the molecule mass, which aligns with Hooke's Law in IR spectroscopy^70^. According to this law, the mass of vibrating molecules is inversely proportional to the frequency of the vibration. Therefore, a lower mass results in a higher frequency of vibration, and consequently, higher wavenumbers. The decrease in the molecular mass may be associated with the onset of degradation, yet peaks are present in FKM-O after 1 year, indicating its resistance to degradation. Comparing the FTIR spectra of FKM-O and FKM-C it can be inferred that there is neither formation of new peaks nor loss of the characteristic peaks of FKM. Additionally, no dehydrofluorination reactions were observed, as indicated by an insignificant change in CH intensity and the preservation of the strongest peak even after 1 year of the interaction of high-temperature oil. Based on these observations, it can be concluded that FKM can sustain harsh hydrocarbon fluid conditions with minimal degradation. For further studies on FKM degradation, refer to our previous work by Wankhede et al.^30,66^.

### Brine absorption in epoxy and FKM

Cured epoxy and FKM samples were tested in American Petroleum Institute (API) standard brine. The API brine used in the test consisted of 2 wt.% Calcium chloride (CaCl_2_) and 8 wt.% sodium chloride (NaCl) dissolved in deionized water^71^. This brine composition is commonly used to simulate brine environments encountered in petroleum industry applications.

The testing procedure involved immersing the epoxy and FKM samples in a hot brine solution at a temperature of 100 °C for 48 h. Furthermore, tests for a longer duration, i.e., 6 months, were performed at room temperature. The percentage change in weight was calculated using Eq. (1). Table 10 shows the percentage weight change in the Epoxy and FKM samples treated in brine at room temperature and at 100 °C for 48 h.

**Table 10:** Summary of percentage weight change in the Epoxy and FKM samples treated in brine at room temperature and 100 °C for 48 h showing negligible degradation of the materials due to exposure from the brine solution.

Material	% Weight change of samples treated in Brine at room temperature for 6 months	% Weight change of samples treated in Brine @100 °C for 48 h
Epoxy	0.31	0.85
FKM	0.22	0.48

The measurements indicate that the percentage weight change of the encapsulation materials in both conditions is less than 1%. Based on this observation, we can conclude that there was negligible degradation of the materials due to exposure to the brine solution.

### Accelerometer performance before and after encapsulation

**Figure 14:**
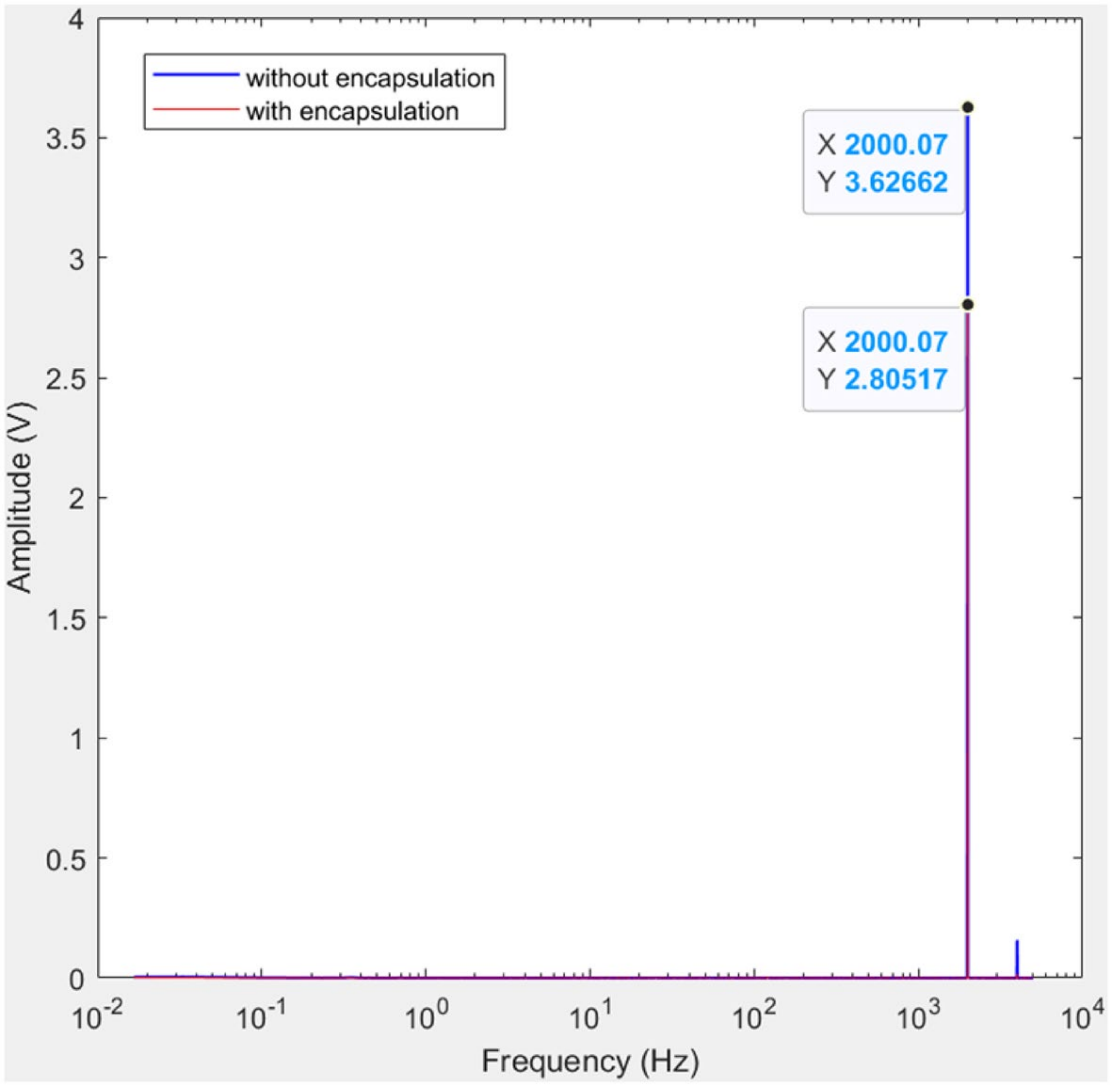
FFT plots show no change in frequency sensed by the accelerometer before and after encapsulation.

Performance evaluations for both accelerometers were conducted under two conditions: one at room temperature without encapsulation and the other immersed in a high-temperature hydrocarbon fluid environment. The accelerometers were subjected to a known input sine wave frequency of 2000 Hz with Vpp of 4 V and the corresponding output frequency was measured by the sensor. Each sensor underwent this test 5 times*. *Table S1 Supplementary information illustrates the output frequency measured by the sensor for the corresponding input frequency before and after encapsulation. From Table S1 supplementary information it can be concluded that the accelerometer was able to capture the given input frequency of 2000 Hz, with a negligible error of 0.07 Hz, both with and without encapsulation. Therefore, the addition of the encapsulation layer did not change the performance of the sensor. To further analyze the sensor performance, a fast Fourier transform (FFT) plot was generated. Figure 14 depicts the FFT plots for the sensor, both before and after encapsulation. Upon examination, it becomes apparent that there is a negligible change in the frequency detected by the accelerometer before and after encapsulation. Specifically, the frequency of 2000 Hz remains constant in both instances. However, the magnitude of vibration decreases for the encapsulated sensor, which is expected due to the presence of the encapsulation material between the vibration source and the sensing surface of the sensor. Following the sensor mounting plot published in API Standard 670, Figure N.34^72^ (referred to Fig. S7 supplementary information), the highest sensitivity can be achieved by stud mounting in which the sensor face is in direct contact with the vibration source. However, this method is suitable for measuring frequencies greater than 10 kHz. Our mounting configuration closely resembles adhesive mounting, where the sensor face is covered with adhesive, preventing direct contact with the vibration source. This method is suitable for frequencies below 10 kHz, which aligns with the requirements of our application. Thus, in our case, adding supplementary encapsulation material for protection against harsh environments does not affect the performance of the sensor and is deemed acceptable. Similar studies were undertaken by Jiang et al.^61^ and Fort et al.^1^ both reporting that encapsulation with epoxy does not affect accelerometer performance in terms of bandwidth and sensitivity.

Further, the sensitivity can be improved by the sensor face open encapsulation design shown in Fig. S8 supplementary information. In this design, the sensing face of the sensor is in direct contact with the vibration source (mounting plate in this study) with the help of a stud surrounded by the encapsulation layers. Direct contact with the vibration source will aid in capturing maximum vibration without loss in sensitivity.

### Leak testing

Leak testing in oil was carried out on three housings:(1) commercial housing CMCP280, (2) side exit sensor housing (3) vertical exit sensor housing. The commercial housing, shown in Fig. 2, is not designed to be leakproof. However, we attempted to enhance its leak resistance by applying high-density thread sealant tape and a rubber washer. Before sealing the housing, we placed oil-absorbing paper inside to detect any oil traces. Initially, all three housings underwent leak tests in water and oil at room temperature for a week. Subsequently, they were submerged in oil at 150 °C for an additional week. No leak was detected in any of the housings for the water and oil leak tests conducted at room temperature. However, within 24 h of submersion at 150 °C, small oil traces were observed in the commercial housing. After 1-week, significant oil presence was observed, as shown in Fig. S9 supplementary information. In contrast, negligible oil traces were found inside the housing developed in this study after a week shown in Fig. S10 supplementary information. The observed leakage in the commercial housing can be attributed to the sealing design. For instance, the neoprene gasket provided with the commercial housing has a maximum operating temperature of 120 °C, making it suitable for intermittent hydrocarbon exposure but unsuitable for continuous use at higher temperatures^73^. Additionally, the positioning of mounting bolts inside the sensor housing may provide a direct pathway for the liquid to enter into a casing. Considering the use of multi-start threads instead of single-start threads could offer a viable solution to achieve leakproofing. Further thermal expansion caused in the various parts like metal plate, bolts, and nuts, rubber gasket in high temperatures potentially may loosen the assembly creating leak paths.

As discussed in previous sections, epoxy and FKM have demonstrated excellent resistance to high-temperature oil. Considering the probable causes for leakage in commercial housing we minimized it using our housing design. However, it is important to address potential manufacturing defects that may arise during the molding process of the epoxy casing, as these defects may create leak paths. Hence, improvements in the manufacturing process of the epoxy casing are required in the future.

## Conclusion

In conclusion, this study has successfully explored the feasibility of utilizing epoxy and FKM as encapsulation materials for prolonged immersion in high-temperature hydrocarbon fluids. The materials endured a rigorous testing period of 10,080 h at 150 °C, during which their performance was meticulously assessed through mass variation tests, microscopic imaging, FTIR spectroscopy, and exposure to API brine solution. Remarkably, negligible degradation was observed, highlighting the robustness of these encapsulation materials.

Furthermore, the study introduced a novel multilayer encapsulation approach employing epoxy and FKM to protect commercial accelerometers. These encapsulated sensors demonstrated their capability to accurately sense vibrations, maintaining acceptable amplitude changes. A comparative analysis between commercial housing and newly developed housing revealed a significant advantage for the latter. While both housings remained leak-free at room temperature, exposure to 150 °C hydrocarbon fluid led to rapid oil ingress in the commercial housing within 24 h, whereas the housing developed in this study displayed minimal traces of oil even after a week. This achievement signifies a breakthrough in encapsulation technology, enabling commercial accelerometers to operate reliably in demanding high-temperature hydrocarbon fluid environments.

To the best of our knowledge, this research represents the first attempt to establish an enclosed packaging solution using epoxy and FKM for commercially available side exit sensors. This innovative encapsulation method holds tremendous potential for various industries, as it enables the secure deployment of commercial accelerometers in high-temperature hydrocarbon fluid settings. Consequently, the data collected by these sensors will empower industries to leverage advanced technologies, such as digital twin modeling, machine learning, and IoT condition monitoring, for early detection and mitigation of potential issues or anomalies. This advancement heralds a new era of precision and efficiency in monitoring and maintaining critical systems in challenging environments, with wide-ranging implications for industry sectors relying on such equipment.

### Supplementary Information

Supplementary Information.

## Data Availability

The datasets used and/or analyzed during the current study are available from the corresponding author upon reasonable request.

## References

[1] Fort, A., Landi, E., Mugnaini, M. & Vignoli, V. Performance of reinforced epoxy resin embedded MEMS accelerometers for IoT condition monitoring. *2023 IEEE International Instrumentation and Measurement Technology Conference (I2MTC)* 01–06 (2023). doi:10.1109/I2MTC53148.2023.10176045.

[2] Yang B, Yang S, Lv Z, Wang F, Olofsson T (2022). Application of digital twins and metaverse in the field of fluid machinery pumps and fans: A review. Sensors.

[3] Kasat P (2023). Developing a digital twin of centrifugal pump for performance evaluation. Mater. Today Proc..

[4] Ambade A, Karnik S, Songchitruksa P, Sinha RR, Gupta S (2021). Electrical submersible pump prognostics and health monitoring using machine learning and natural language processing. OnePetro.

[5] Sircar A, Yadav K, Rayavarapu K, Bist N, Oza H (2021). Application of machine learning and artificial intelligence in oil and gas industry. Pet. Res..

[6] Corley, J. E. Vibrational problems of large vertical pumps and motors. *Proceedings of the 9th Turbomachinery Symposium* (Texas A&M University. Gas Turbine Laboratories). doi:10.21423/R1TH5S.(1980)

[7] Trout, J., Vibration Analysis Explained | Reliable Plant. Noria Corporation https://www.reliableplant.com/vibration-analysis-31569.(2023)

[8] Lakal N (2022). Sensing technologies for condition monitoring of oil pump in harsh environment. Sens. Actuators Phys..

[9] Vertical Industrial Can-Type Pumps | Goulds Pumps. https://www.gouldspumps.com/en-US/Products/VIC/.

[10] What is an IP rating. *Samsung au* https://www.samsung.com/au/support/mobile-devices/water-and-dust-protection-ip68-rating/.

[11] Christman, M. Understanding Ingress Protection Ratings. https://www.pcb.com/https://www.pcb.com/nx/search-results?q=understanding%20ingress%20protection%20ratings.

[12] Sinha A, Joshi YK (2011). Downhole electronics cooling using a thermoelectric device and heat exchanger arrangement. J. Electron. Packag..

[13] Downhole Temperature - an overview | ScienceDirect Topics. https://www.sciencedirect.com/topics/engineering/downhole-temperature.

[14] Bently Nevada 43217 / 37442 Accelerometer Mounting Kit | Instrumart. https://www.instrumart.com/products/47008/bently-nevada-43217-37442-accelerometer-mounting-kit.

[15] STI Vibration Monitoring Inc. www.stiweb.comhttps://www.stiweb.com/product_p/cmcp280.htm.

[16] MH152–1A Top Exit Sensor Protector | CTC. https://ctconline.com/products/ctc-line/mounting-hardware/sensor-protectors?prd=MH152-1A.

[17] MH148–1A Side Exit Sensor Protector | CTC. https://www.ctconline.com/products/ctc-line/mounting-hardware/sensor-protectors?prd=MH148-1A.

[18] Ma Y, Sui Y, Li T, Gianchandani YB (2015). A submillimeter package for microsystems in high-pressure and high-salinity downhole environments. J. Microelectromech. Syst..

[19] Choi M (2017). Autonomous microsystems for downhole applications: Design challenges, current state, and initial test results. Sensors.

[20] Seren HR (2018). An untethered sensor platform for logging vertical wells. IEEE Trans. Instrum. Meas..

[21] Buzi E, Seren HR, Deffenbaugh M, Turner R, Ssafwany A (2020). Sensor ball: An autonomous untethered logging platform. OnePetro.

[22] Sui Y (2021). An autonomous environmental logging microsystem (ELM) for harsh environments. IEEE Sens. J..

[23] Epoxy Adhesives. *Handbook of Adhesive Technology* (ed. Rudawska, A.) (CRC Press, 2017).

[24] Ishii T, Tsuyuno N, Sato T, Masuda M (2006). Corrosion studies of copper and aluminum interconnects exposed to automotive oils. IEEE Trans. Compon. Packag. Technol..

[25] Singh N, Sinha SK (2022). Effects of soaking in water, base oil, ionic liquid, and grease of an Epoxy/UHMWPE/MoS2 composite on mechanical and tribological properties. J. Tribol..

[26] Kim C (2018). Effect of microscale oil penetration on mechanical and chemical properties of carbon fiber-reinforced epoxy composites. J. Ind. Eng. Chem..

[27] Wnuk VP, Mendez A, Ferguson S, Graver T (2005). Process for mounting and packaging of fiber Bragg grating strain sensors for use in harsh environment applications. Smart Struct..

[28] Linz T (2008). Embroidered interconnections and encapsulation for electronics in textiles for wearable electronics applications. Adv. Sci. Technol..

[29] Birkelund K, Nørgaard L, Thomsen EV (2011). Enhanced polymeric encapsulation for MEMS based multi sensors for fisheries research. Sens. Actuators Phys..

[30] Wankhede S, Du X, Alshehri A, Brashler K, Turcan D (2023). Encapsulating and inkjet-printing electronics on flexible substrates for harsh environment. Am. Soc. Mech. Eng. Digit. Collect..

[31] Ma F, Guo F, Zhou H, Wei J, Zhen W (2014). A fiber bragg grating temperature sensor with high pressure-resistance applied in down-hole. Adv. Mater. Res..

[32] Boeser, F., Ordonez, J. S., Schuettler, M., Stieglitz, T. & Plachta, D. T. T. Non-hermetic encapsulation for implantable electronic devices based on epoxy. *37th Annual International Conference of the IEEE Engineering in Medicine and Biology Society (EMBC)* 809–812 (2015). doi:10.1109/EMBC.2015.7318485. 10.1109/EMBC.2015.7318485 26736385

[33] Stosur M, Sowa K, Piasecki W, Płatek R, Balcerek P (2017). Encapsulation of power electronics components for operation in harsh environments. Arch. Electr. Eng..

[34] Spanier, M. et al. (2022). A novel hermetic encapsulation approach for the protection of electronics in harsh environments. In: 2022 IEEE 9th Electronics System-Integration Technology Conference (ESTC) 145–149 doi:10.1109/ESTC55720.2022.9939521.

[35] Phua EJR (2018). Novel high temperature polymeric encapsulation material for extreme environment electronics packaging. Mater. Des..

[36] Tay YS (2023). Ruggedized sensor packaging with advanced die attach and encapsulation material for harsh environment. Microelectron. Reliab..

[37] ASTM D1418–21a - Standard Practice for Rubber and Rubber Latices - Nomenclature. https://webstore.ansi.org/standards/astm/astmd141821a.

[38] Viton Rubber Seal | Compression Molding Services. https://qualiformrubbermolding.com/products/rubber-products/viton-rubber-seal/.

[39] Fluoroelastomers, FKM, FPM Info - Pelseal Technologies. https://www.pelseal.com/why-fluoroelastomers.

[40] Sharma G, Klintberg L, Hjort K (2011). Viton-based fluoroelastomer microfluidics. J. Micromech. Microeng..

[41] Vellaluru N (2022). Autonomous sensing microsystem with H2S compatible package and enhanced buoyancy for downhole monitoring. SPE J..

[42] Monshi MM, Camara J-S, Bhardwaj S, Volakis JL, Raj PM, Monshi MM (2020). High-density embedded electronics in textiles with 3D flex package transfer. 2020 IEEE 70th Electronic Components and Technology Conference (ECTC).

[43] Seo J, Ha J, Lee B, Kim H, Hong Y (2020). Fluoroelastomer encapsulation for enhanced reliability of solution-processed carbon nanotube thin-film transistors. Thin Solid Films.

[44] Takahashi Y, Takahashi T, Abe T, Noma H, Sohgawa M (2022). Tactile sensor with microcantilevers embedded in fluoroelastomer/PDMS for physical and chemical resistance. Electron. Commun. Jpn..

[45] FKM - Viton, Fluorel Rubber. *Rahco Rubber* https://rahco-rubber.com/materials/fkm-viton-fluorel-rubber/.

[46] CRG: Properties of Viton. https://chemours-util.my.salesforce-sites.com/CRG_VitonProperties.

[47] DuPont Mobility and Materials Kalrez® 4079 FFKM Perfluoroelastomer. https://www.matweb.com/search/datasheet.aspx?matguid=0914d75467f248cc8a4b319c27df5fa3&n=1&ckck=1.

[48] FFKM vs FKM | Perfluoroelastomer Properties | TRP. *TRP Polymer Solutions* https://trp.co.uk/the-material-properties-of-perfluoroelastomer-ffkm/ (2016).

[49] EPDM - Ethylene Propylene Rubber. *Rahco Rubber* https://rahco-rubber.com/materials/epdm-ethylene-propylene-rubber/.

[50] Buna N - Properties and Uses. *Our Education | Best Coaching Institutes Colleges Rank* https://blog.oureducation.in/buna-n-properties-and-uses/ (2013).

[51] Buna-N (NBR): What is it and When to Use it. *Techno Ad* https://www.technoad.com/buna-n-nbr/ (2022).

[52] Neoprene Rubber. *Rahco Rubber* https://rahco-rubber.com/materials/cr-neoprene-rubber/.

[53] Std, 12 in x 12 in, Silicone Sheet. *Grainger* https://www.grainger.com/product/GRAINGER-APPROVED-Silicone-Sheet-Std-1MVR1.

[54] VMQ - Silicone Rubber. *Rahco Rubber* https://rahco-rubber.com/materials/vmq-silicone-rubber/.

[55] Du X (2022). A review of inkjet printing technology for personalized-healthcare wearable devices. J. Mater. Chem. C.

[56] PC-Fahrenheit^TM^ – Protective Coating Company. https://www.pcepoxy.com/products/emergency-repair/pc-fahrenheit/.

[57] LOCTITE® STYCAST 2762FT. https://www.henkel-adhesives.com/us/en/product/potting-compounds/loctite_stycast_2762ft0.html.

[58] Model EXHT622B01 | PCB Piezotronics. https://www.pcb.com/products?m=exht622b01.

[59] Model 625B01 | PCB Piezotronics. https://www.pcb.com/products?m=625b01.

[60] L. Da Rocha, M., De O. Leite, M. C., P. Da C. Ferreira, E., D. Melo, J. D. & C. Barbosa, A. P. Accelerated aging effects in composites used as repair for pipes in oil industry. *Polym. Compos.* **42**, 5918–5929 (2021).

[61] Jiang Y, Du M, Luo L, Li X (2004). Simulation of the potting effect on the high-G MEMS accelerometer. J. Electron. Mater..

[62] ASM Material Data Sheet. https://asm.matweb.com/search/SpecificMaterial.asp?bassnum=mq316a.

[63] Wadsworth, S. What Type of Epoxy Are You Using and Why? *Southern Industrial Supply* https://southern-industrial.com/blogs/news/what-type-of-epoxy-are-you-using-and-why (2018).

[64] Sukanto H, Raharjo WW, Ariawan D, Triyono J, Kaavesina M (2021). Epoxy resins thermosetting for mechanical engineering. Open Eng..

[65] Bisphenol F Epoxy Resins | Business & Products. *DIC Corporation* https://www.dic-global.com/en/products/epoxy/bpf/.

[66] Wankhede SP, Alshehri AH, Du X (2023). Encapsulating and inkjet-printing flexible conductive patterns on a fluoroelastomer for harsh hydrocarbon fluid environments. J. Mater. Chem. C.

[67] Chemical Compatibility Chart | Applications. *Jehbco Silicones* https://jehbco.com.au/products/chemical-compatibility-chart/.

[68] Lee JN, Park C, Whitesides GM (2003). Solvent compatibility of poly(dimethylsiloxane)-based microfluidic devices. Anal. Chem..

[69] Wang Q-L (2020). Accelerated aging behaviors and mechanism of fluoroelastomer in lubricating oil medium. Chin. J. Polym. Sci..

[70] Schaller, C. 3.3: Some Subtle Points of IR Spectroscopy in *Structure & Reactivity II: Purification and Spectroscopy* (Chemistry LibreTexts, 2019). https://chem.libretexts.org/Bookshelves/General_Chemistry/Book%3A_Structure_and_Reactivity_in_Organic_Biological_and_Inorganic_Chemistry_(Schaller)/Structure_and_Reactivity_in_Organic_Biological_and_Inorganic_Chemistry_II%3A_Practical_Aspects_of_Structure_-_Purification_and_Spectroscopy/03%3A_Infrared_Spectroscopy/3.03%3A_Some_Subtle_Points_of_IR_Spectroscopy (2019).

[71] Chapman D, Trybula W (2012). Meeting the challenges of oilfield exploration using intelligent micro and nano-scale sensors. IEEE Int. Conf. Nanotechnol. (IEEE-NANO).

[72] API Standard 670 Machinery Protection Systems. American Petroleum Institute. https://www.api.org/products-and-services/standards/purchase (2014).

[73] Hoffman, J. Which Materials are Best for Oil Resistance? - Blog | Rubber Articles | Timco Rubber. https://www.timcorubber.com/blog/archive/which-materials-are-best-for-oil-resistance (2022).

