# Supplementary material for: Encapsulating commercial accelerometers with epoxy and fluoroelastomer for harsh hydrocarbon fluid environment
Source: Sci Rep. 2023 Nov 13;13:19815. doi: 10.1038/s41598-023-46781-x (PMC10643642; doi:10.1038/s41598-023-46781-x)
Supplement: Supplementary file 1 — Supplementary Information. [file 41598_2023_46781_MOESM1_ESM.docx]

Supplementary information

**Encapsulating Commercial Accelerometers with Epoxy and Fluoroelastomer for Harsh Hydrocarbon Fluid Environment**

Sahil P. Wankhede

Department of Mechanical Engineering, University of Massachusetts Amherst, MA 01003, U.S.A

Center for Personalized Health Monitoring, (CPHM), Institute for Applied Life Sciences (IALS), MA 01003, U.S.A

swankhede@umass.edu

Xian Du^*^

Department of Mechanical Engineering, University of Massachusetts Amherst, MA 01003, U.S.A

Center for Personalized Health Monitoring, (CPHM), Institute for Applied Life Sciences (IALS), MA 01003, U.S.A

xiandu@umass.edu

Keith W Brashler

Saudi Arabian Oil Company (Saudi Aramco), Dhahran 31311, Saudi Arabia

keith.brashler@aramco.com

Mohammad Ba'adani

Saudi Arabian Oil Company (Saudi Aramco), Dhahran 31311, Saudi Arabia

mohammad.baadani@aramco.com

Doru C Turcan

Saudi Arabian Oil Company (Saudi Aramco), Dhahran 31311, Saudi Arabia

[doru.turcan@aramco.com](mailto:doru.turcan@aramco.com)

Ali H. Alshehri

Saudi Arabian Oil Company (Saudi Aramco), Dhahran 31311, Saudi Arabia

ali.alshehri@aramco.com

Kamal Youcef-Toumi

Massachusetts Institute of Technology, 77 Massachusetts Avenue Cambridge, Massachusetts 02139, U.S.A.

youcef@mit.edu

^*^ Corresponding author

Xian Du, Department of Mechanical Engineering, University of Massachusetts Amherst, MA 01003, U.S.A

Center for Personalized Health Monitoring, (CPHM), Institute for Applied Life Sciences (IALS), MA 01003, U.S.A

xiandu@umass.edu


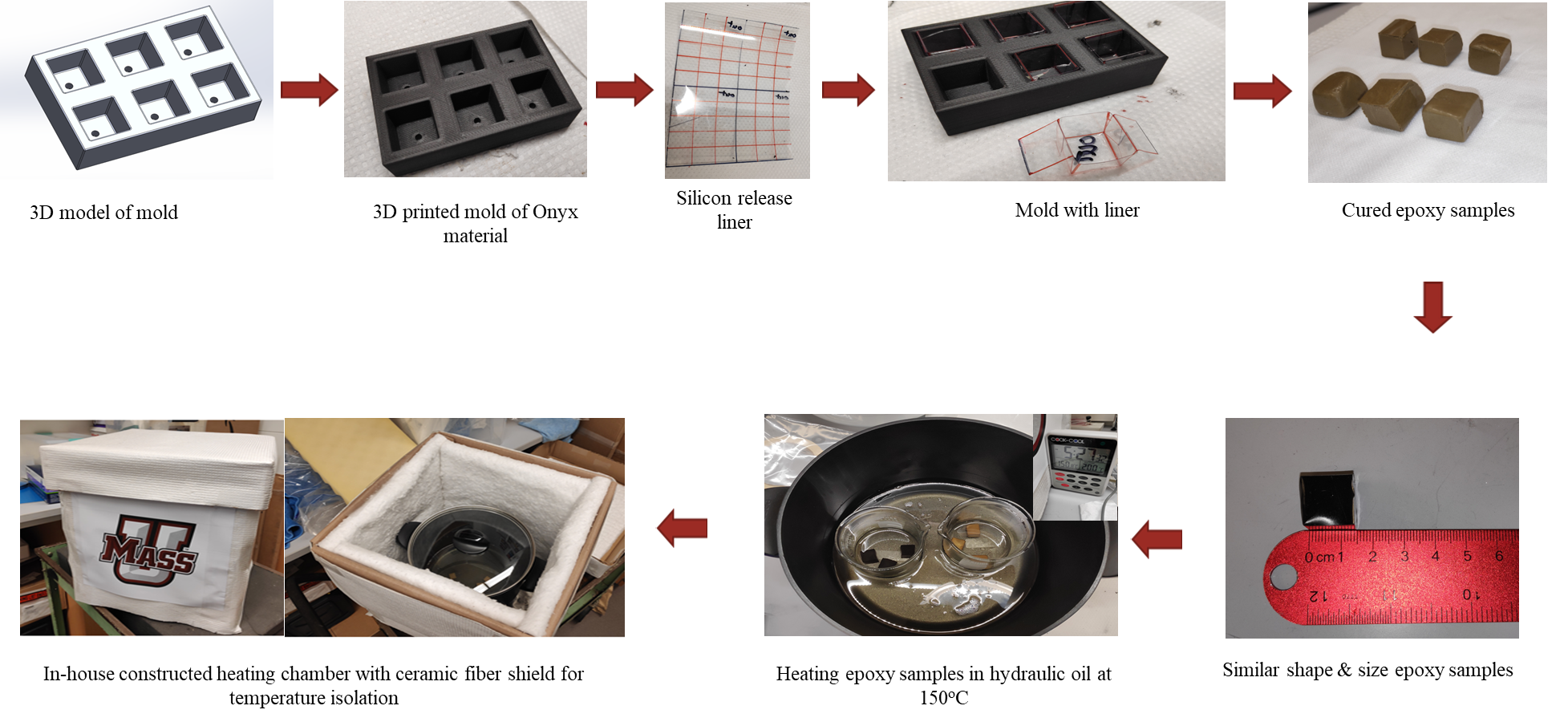


Figure S1. Epoxy PC Fahrenheit and LOCTITE STYCAST 2762FT specimen preparation process flow.

158 MPa

Figure S2. Epoxy LOCTITE STYCAST 2762FT compression stress vs strain curve showing the ultimate compression strength of the epoxy 158 MPa.


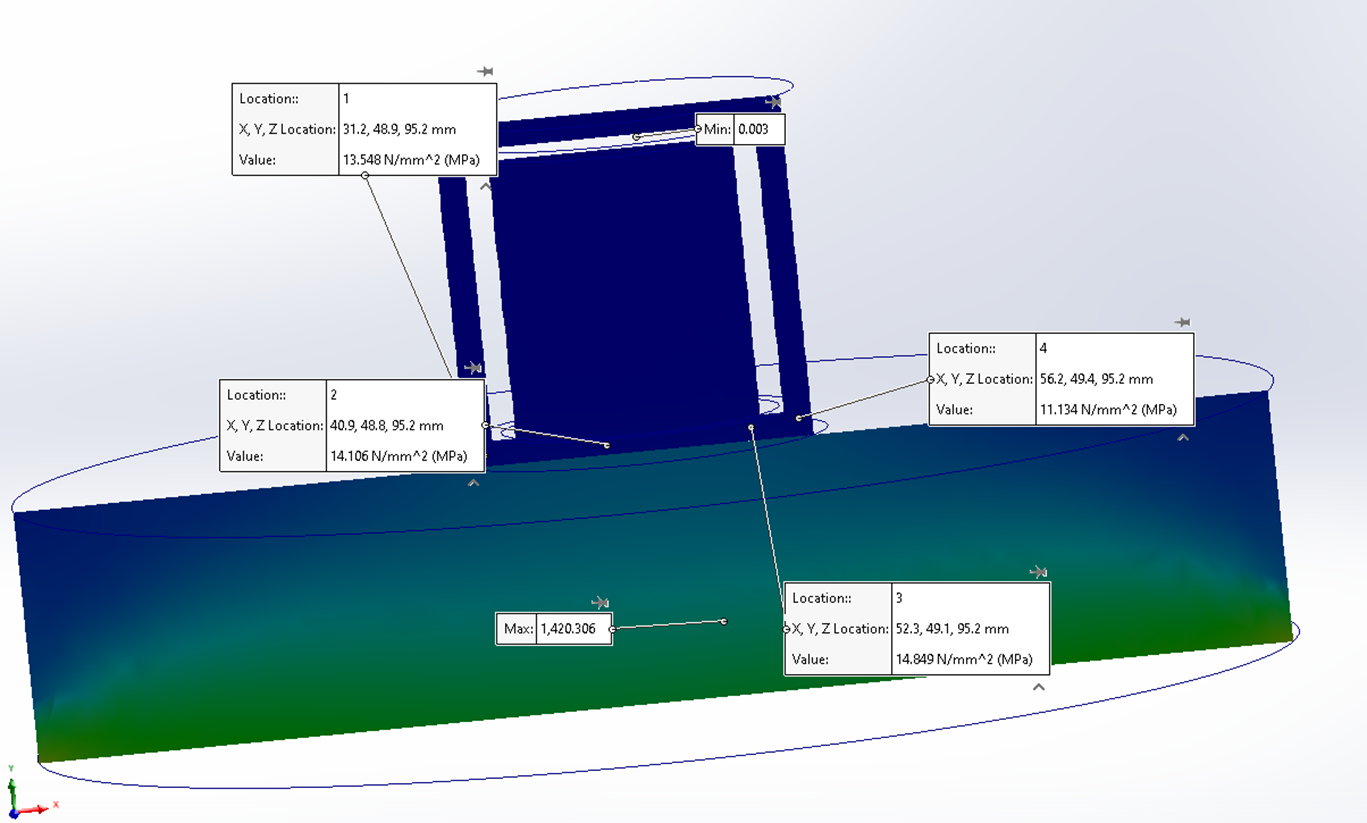


Figure S3. Stress was generated in the epoxy LOCTITE STYCAST 2762FT at the intersection zone between the sensor and the epoxy.

The average stress generated in the epoxy at the intersection zone was found to be 13.5 MPa, which is less than 158 MPa.


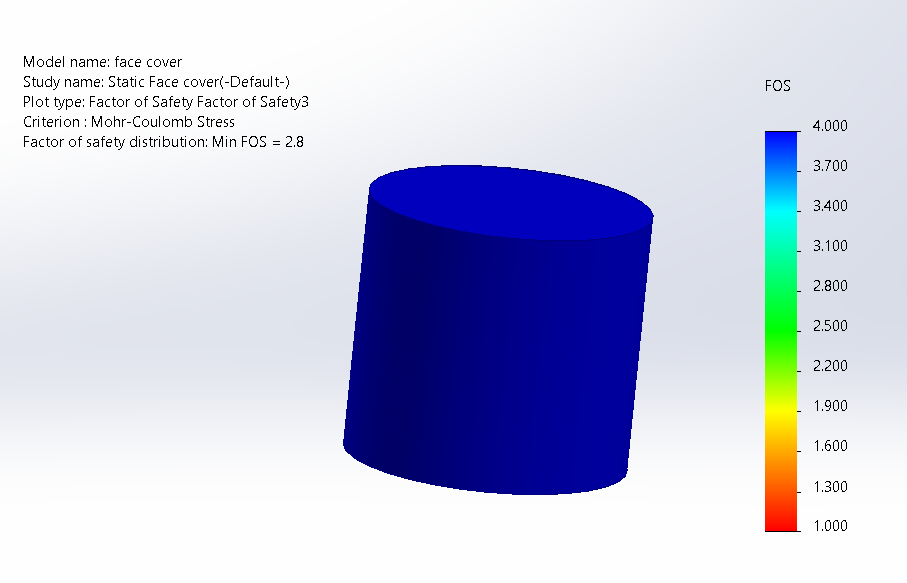


Figure S4. Factor of safety of 2.8 considering Mohr-Coulomb Theory


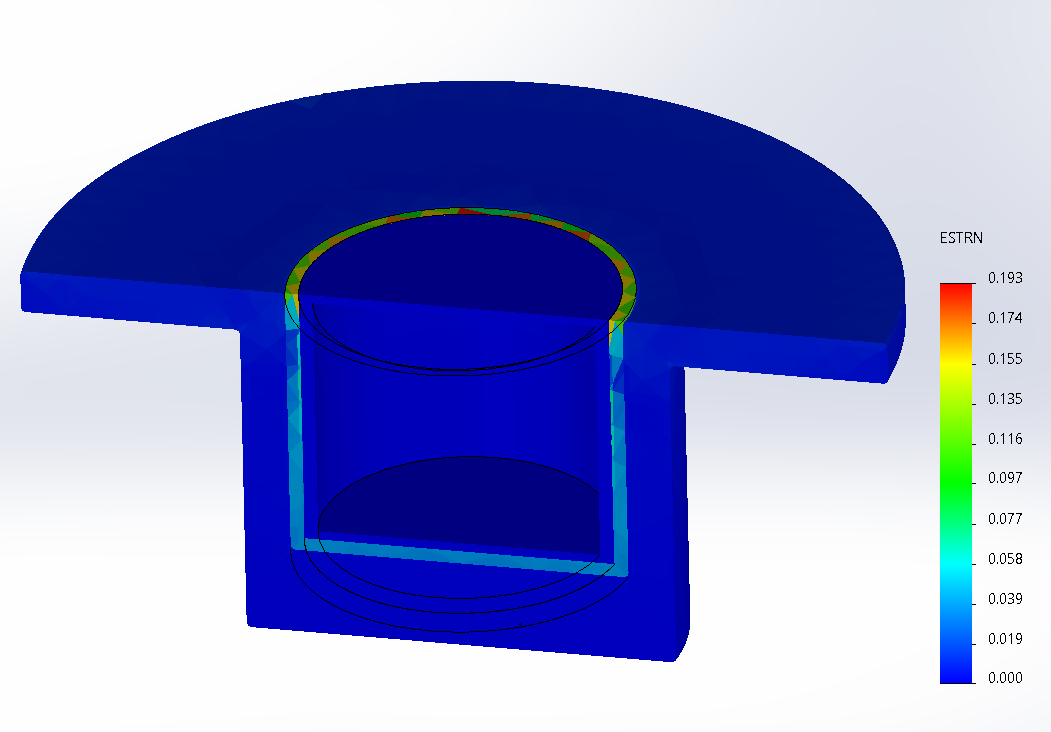


(a)


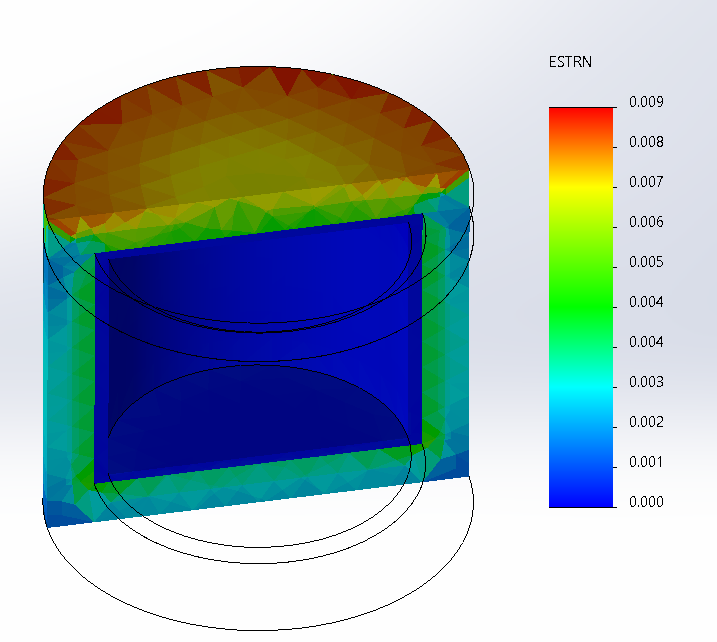


(b)

Figure S5. Strain distribution in the encapsulated structure (a) With FKM layer (b) Without FKM.

When FKM is incorporated, the thermal strain developed in the model is not transferred to the epoxy and steel body.

**
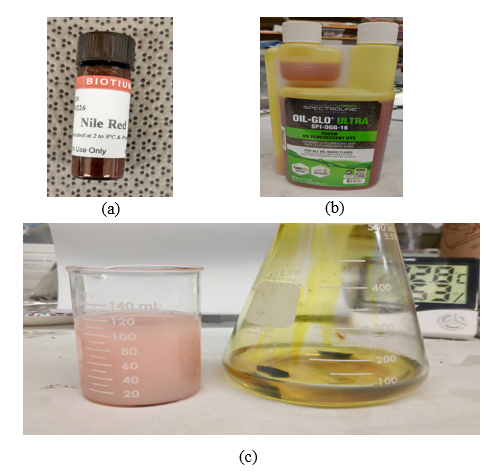
**

Figure S6. Epoxy cube submerged in Nile Red and Oil Glo solution mixed in hydraulic oil. . (a) Nile Red (b) Oil -Glo (c) Nile red mixture in Ethanol and Hydraulic oil (left); Oil Glo mixed with Hydraulic oil (right)

Solution preparation recipe:

Nile Red solution: 1mg of Nile red powder was mixed in 100 ml ethanol and stored at 2-8°C. Further, 10 ml of this solution was mixed with 100 ml of hydraulic oil.

Oil Glo solution: Mixed 0.1 ml of Oil Glo in 100 ml of hydraulic oil.

**
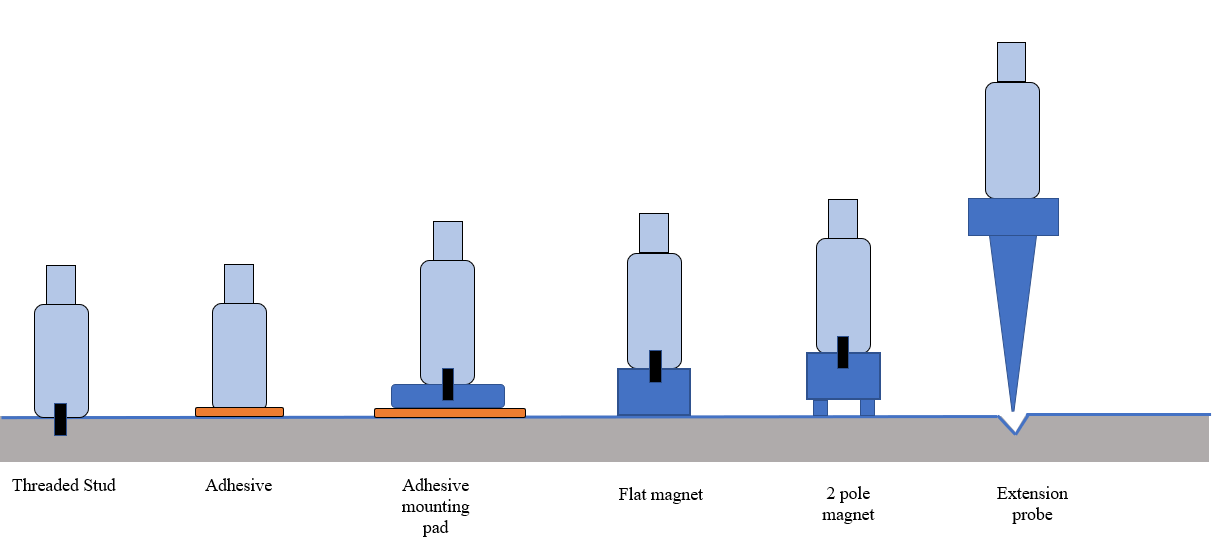
**

**
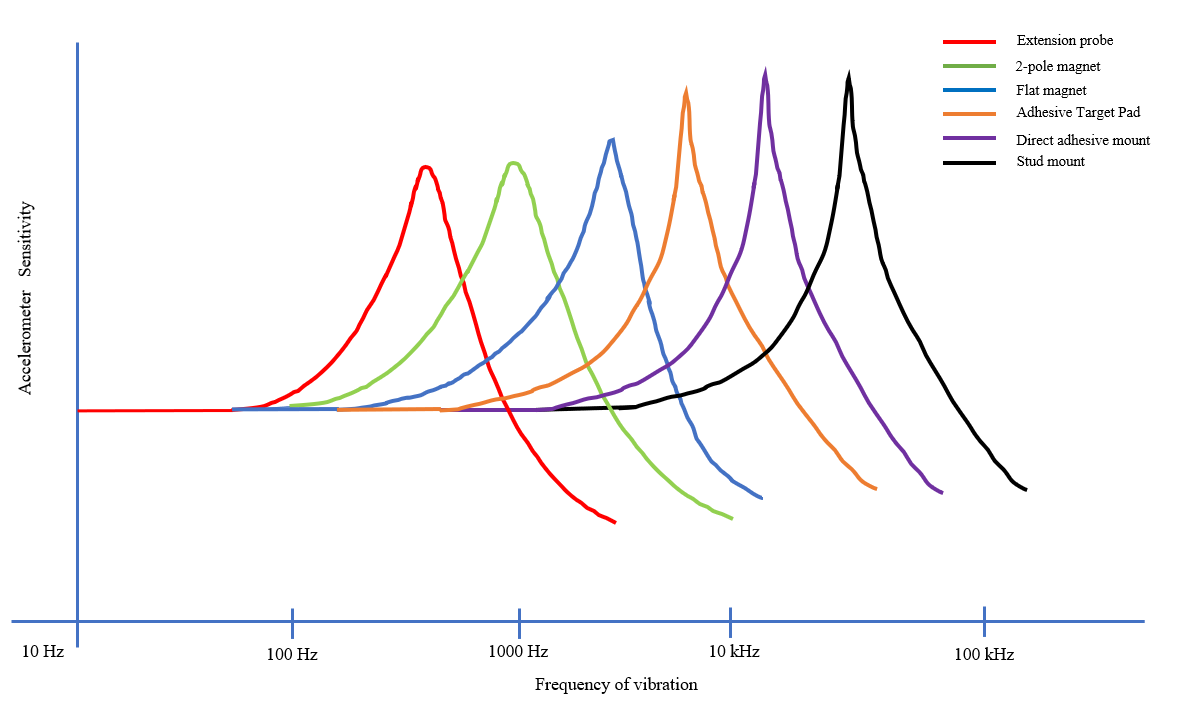
**

Figures S7. Change of accelerometer sensitivity for various mounting methods. Redrawn from API Standard 670, Figure N.34^1^


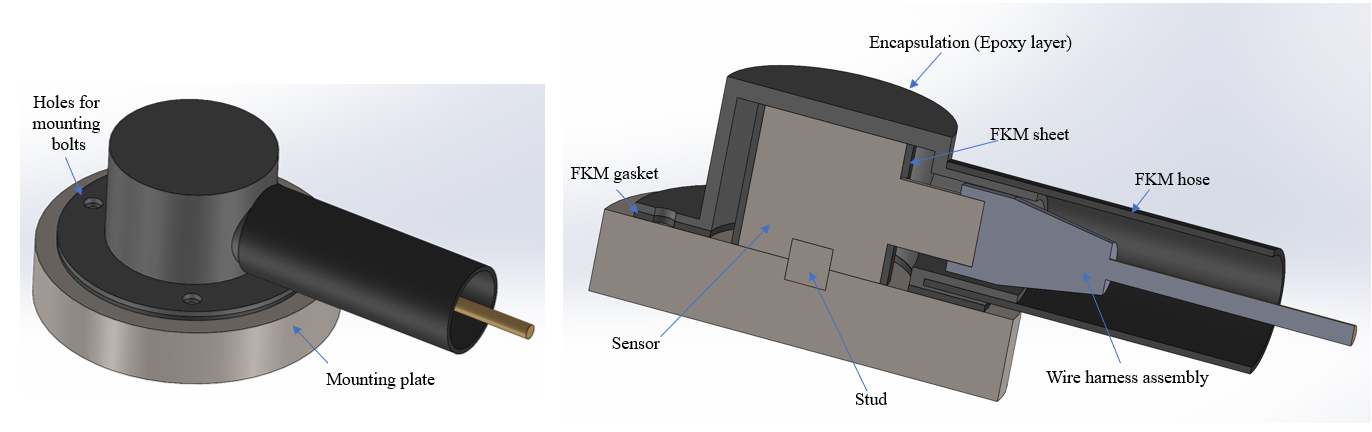


Figure S8. Proposed model of multilayer encapsulation structure for side exit sensor with sensor face in direct contact with vibration source to improve its sensitivity.


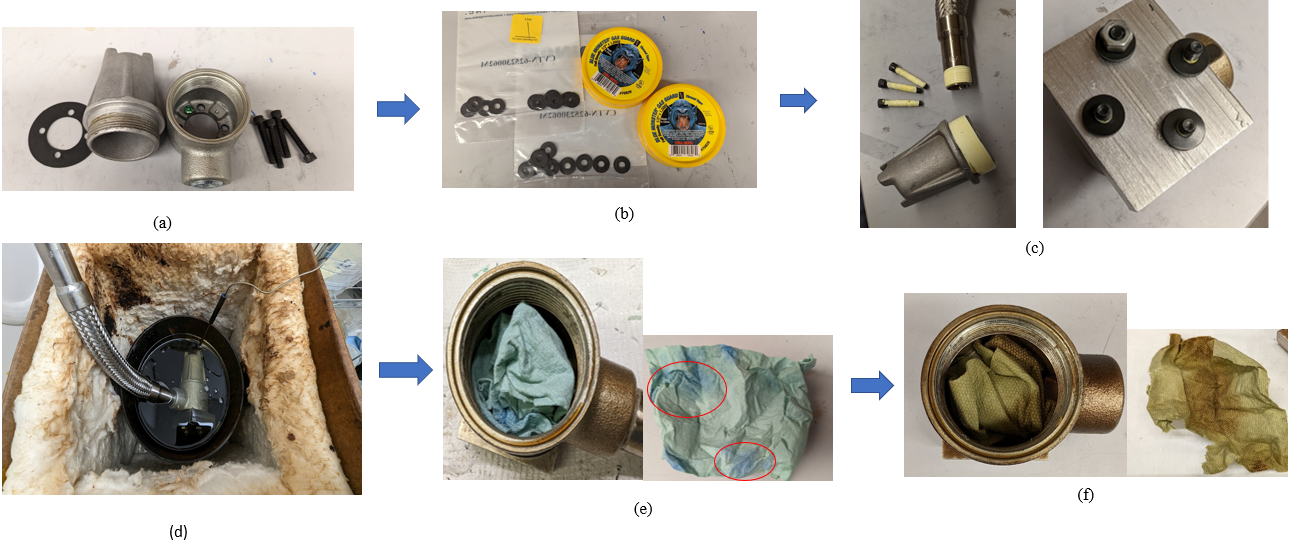


Figure S9. Commercial housing leak test in oil at 150°C. (a) Disassembled commercial housing (b) High-density thread sealant and Viton washer for high temperature (c) Applying thread sealant and washer to housing and hose (d) Housing assembly submerged in oil (e) Small oil traces found after 24 hours at 150°C (f) Significant oil traces found after 1 week at 150°C.


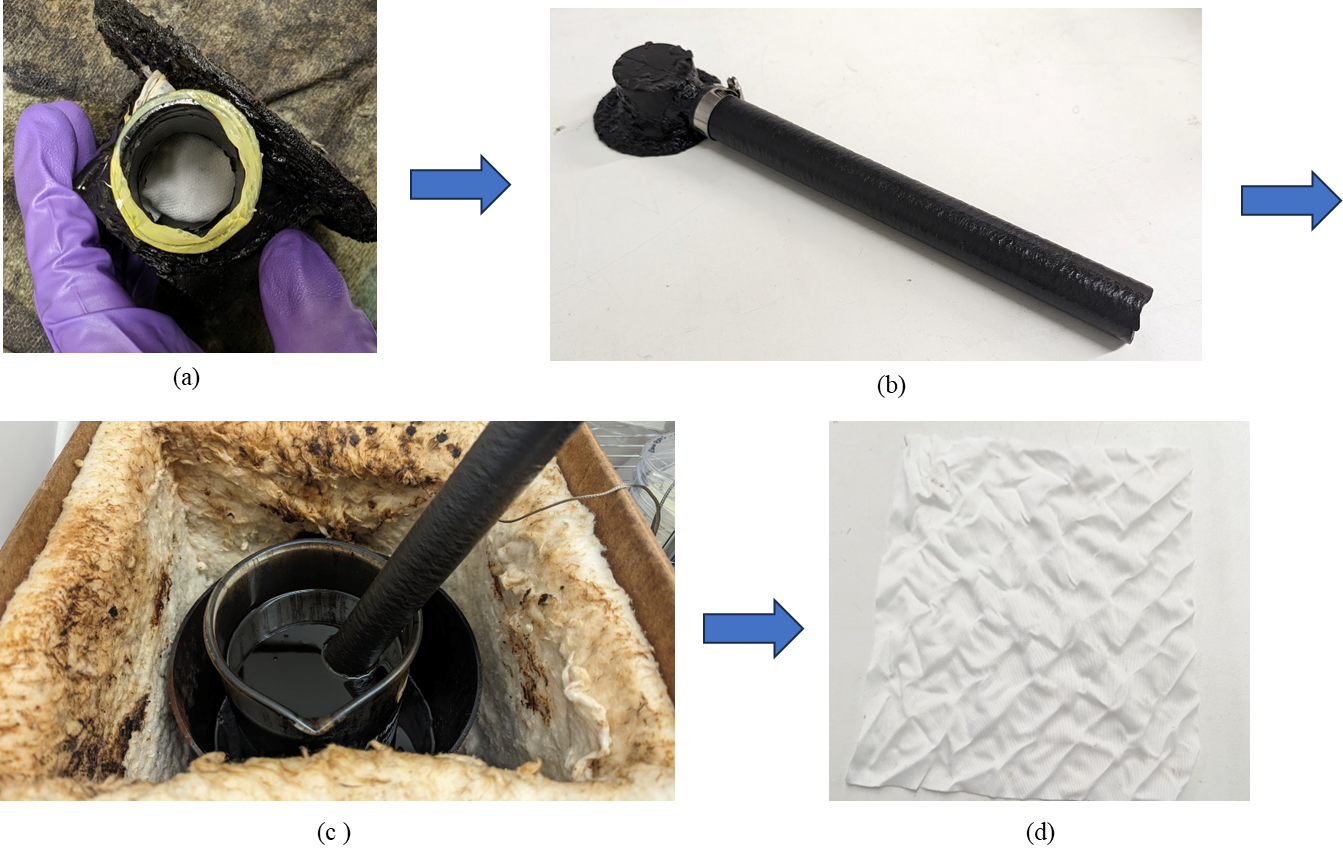


Figure S10. Leak test in oil at 150°C of housing developed in this work showing negligible oil traces (a) Test cloth inserted into the housing (b) assembly of the housing and FKM hose (c) Housing assembly submerged in the oil bath at 150°C.

(d) Test cloth showing no oil traces

Table S1. Measurement results by the accelerometer showing no change in frequency sensed by the accelerometer before after encapsulation.

| Sr. No. | Output frequency (Hz) (without encapsulation)  EXHT622B01 | Output frequency (Hz) (without encapsulation)  625B01 | Output frequency (Hz)  (with encapsulation)  EXHT622B01 | Output frequency (Hz) (with encapsulation)  625B01 |
| --- | --- | --- | --- | --- |
| 1 | 2000.07 | 2000.07 | 2000.07 | 2000.07 |
| 2 | 2000.07 | 2000.07 | 2000.07 | 2000.07 |
| 3 | 2000.07 | 2000.07 | 2000.07 | 2000.07 |
| 4 | 2000.07 | 2000.07 | 2000.07 | 2000.07 |
| 5 | 2000.07 | 2000.07 | 2000.07 | 2000.07 |
| Avg | 2000.07 | 2000.07 | 2000.07 | 2000.07 |

**Reference**

1. API Standard 670 Machinery Protection Systems, DOI https://www.api.org/products-and-services/standards/purchase., 2014
